# Phenotypic and functional changes to porcine monocyte-derived macrophages triggered by IL-1β and its receptor antagonist IL-1Ra

**DOI:** 10.3389/fimmu.2026.1869718

**Published:** 2026-08-03

**Authors:** Emanuela Giaconi, Samanta Mecocci, Susanna Zinellu, Valentina Margarita, Sarah Morrone, Nicolò Columbano, Luisa Bogliolo, Sandra Barroso-Arévalo, Katia Cappelli, Giulia Franzoni

**Affiliations:** 1 Department of Animal Health, Istituto Zooprofilattico Sperimentale della Sardegna, Sassari, Italy; 2 Department of Veterinary Medicine, University of Perugia, Perugia, Italy; 3 Department of Innovation in Biological, Agro-Food and Forest System (DIFAB), University of Tuscia, Viterbo, Italy; 4 Department of Biomedical Science, University of Sassari, Sassari, SS, Italy; 5 Department of Veterinary Medicine, University of Sassari, Sassari, SS, Italy; 6 Department of Medicine and Animal Surgery, Faculty of Veterinary Medicine, Complutense University of Madrid, Madrid, Spain

**Keywords:** cytokines, domestic pigs, innate immunity, macrophages, surface markers

## Abstract

Pigs are attracting increasing attention as a large-animal model with translational relevance in biomedical research. They share several immunological similarities with humans, although the pig immune system has not been fully characterized yet, also at the level of macrophage polarization. A better understanding of porcine macrophage polarization could improve translational studies, allowing a better interpretation of *in vitro* and *in vivo* results. In this study, we performed a detailed characterization of the impact of two poorly studied cytokines [interleukin-1β (IL-1β) and its receptor antagonist IL-1Ra] on porcine monocyte-derived macrophage (moMФ) phenotype and functions. The effects of these IL-1 family members were investigated through microscopy, flow cytometry, enzyme-linked immunosorbent assay (ELISA), and gene expression studies. We observed that stimulation with IL-1β increased CD14, SLA-I, and SLA-II DR expression, and enhanced the release of pro-inflammatory cytokines, particularly IL-6, IL-12, and IL-1α at higher concentrations, and the expression of several antiviral and pro-inflammatory genes, including *JUN*, *FOS*, and *MAP3K1*. The concomitant release of IL-10 and IL-1Ra suggests that IL-1β stimulation may also activate compensatory regulatory feedback mechanisms aimed at limiting excessive inflammation. In contrast, IL-1Ra had limited effects on surface marker expression but increased IL-10 release. A small but significant release of IL-1α was also observed, as well as a trend toward enhanced *IL1B* expression at 24 h, suggesting a potential regulatory interaction within the IL-1 axis. MoMФ stimulated with IL-1Ra was also able to release high levels of IL-12 in response to stimulation with a TLR-3 ligand [polyinosinic–polycytidylic acid (poly I:C)], with an intensity comparable to moMФ and moMФ stimulated with IL-1β. Our data suggest that porcine macrophages attempt to maintain a balance between IL-1β and IL-1Ra, as a defense mechanism to control inflammation, by counteracting each other’s activity. Overall, in this work, we provided a better understanding of porcine macrophage polarization, which could benefit translational studies using this large-animal model.

## 1 Introduction

Macrophages are innate immune cells and one of the first-line defenses against pathogens and tumor cells (1). These cells not only play a key role in immune responses to invading pathogens but also are involved in clearing senescent cells and cellular debris and repairing tissues after inflammation (1, 2). Macrophages are indeed characterized by strong plasticity: they can acquire distinct functional characteristics in response to changes in the surrounding micro-milieu and the immunological context (1, 3). The two antithetic extremes of activation states are represented by classically activated (M1) macrophages, with increased anti-microbial or antitumor activities, and alternatively activated (M2) macrophages, associated with mechanisms of immunosuppression and wound repair (3, 4).

In humans and mice, M2 macrophages have been generated *in vitro* by exposure to interleukin-4 (IL-4) and/or IL-13, whereas classical macrophage activation (M1) has been achieved *in vitro* by exposure to interferon-γ (IFN-γ) and lipopolysaccharide (LPS) (or another TNF inducer) (4, 5). This simplistic view was subsequently refined with alternative activated macrophages being divided into three subsets: M2a (generated *in vitro* by exposure to IL-4 or IL-13), M2b (generated *in vitro* by exposure to immune complexes with IL-1β or LPS), and M2c (generated *in vitro* by exposure to IL-10, TGF-β, or glucocorticoids) (4, 5). More recently, it was discovered that exposure to diverse activators can lead to unique phenotypes; thus, it was suggested to opt for a nomenclature based on the activator/s used (6).

Each species has its own peculiarities, including pigs. Pigs share several anatomical, physiological, and immunological similarities with humans; thus, they have become one of the main close-to-human models in biomedical research (7, 8). They have been used in preclinical evaluation of vaccine candidates and therapeutics (9, 10), preclinical toxicologic testing of pharmaceuticals or other chemicals (7), and nanomedicine-based studies (11, 12). Nevertheless, the pig immune system has not been fully characterized yet, also at the level of macrophage polarization. Previous studies reported that porcine macrophage plasticity was broadly comparable to human and murine macrophages, but some peculiarities were highlighted (13–15). In particular, we reported that both IL-4 and IL-10 did not trigger the expression/release of IL-10, whereas we observed an enhanced expression of IL-18 (14, 15). In parallel, stimulation with IFN-γ and LPS polarized porcine monocyte-derived macrophages (moMΦ) toward a pro-inflammatory phenotype, but a significant IL-1Ra response was detected (15). Despite the interesting peculiarities observed in pigs in the modulation of IL-1 family members, especially IL-1Ra, the effects of this antagonist receptor and its agonist IL-1β (the prototypical IL-1 cytokine) on porcine macrophage phenotype and functionality remain largely unexplored. Therefore, this study aimed to address this knowledge gap by investigating the effects of IL-1β and IL-1Ra on porcine moMФ.

In mammals, the IL-1 superfamily is composed of IL-1β and other related cytokines, some with pro-inflammatory activities (IL-1α, IL-1β, IL-18, IL-33, IL-36α, IL-36β, and IL-36γ) and others with antagonistic or inhibitory activities (IL-1Ra, IL-36Ra, IL-37, and IL-38) (16). IL-1β is a key pro-inflammatory cytokine, which contributes to defensive responses by promoting and amplifying inflammation (17). Monocytes and macrophages are the main source of this cytokine, but IL-1β is also released by natural killer (NK) cells, B cells, dendritic cells (DCs), fibroblasts, and epithelial cells (18). It is synthesized as a leaderless precursor, or from an inactive IL-1β precursor, which must be cleaved by inflammasome-activated caspase-1 (18). Microbial products sensed through pattern-recognition receptors, including TLRs, typically induce *IL1B* transcription and pro-IL-1β synthesis; secretion of mature IL-1β generally requires an additional processing step, often mediated by inflammasome-associated caspase-1 or alternative proteases (19). IL-1β promotes fever, facilitating lymphocyte functions, which, in mammals, are indeed more pronounced at temperatures above 37 °C. Overall, IL-1β created an unfavorable environment for pathogens (17). The IL-1 system must be tightly controlled, to prevent potentially pathological over-response to stressors. One of the control mechanisms is the release of receptor antagonists, such as IL-1Ra (20). IL-1Ra binds the receptor IL-1R1 with higher affinity than that of IL-1α or IL-1β, but it does not trigger the activation of the IL-1 signaling, blocking further IL-1 activity and preventing the development of exacerbated inflammatory response (18, 20).

We hypothesized that IL-1β and IL-1Ra induce distinct and opposite phenotypic and functional programs in porcine moMΦ, and in this study, we performed a detailed characterization of porcine macrophages following exposure to IL-1β and its receptor antagonist IL-1Ra. The effects of these two interleukins on porcine moMΦ were investigated using a vast array of techniques, including flow cytometry, multiplex enzyme-linked immunosorbent assay (ELISA), and gene expression analysis. A better understanding of porcine macrophage polarization could improve translational studies using pigs as a close-to-human model, allowing a better interpretation of *in vitro* and *in vivo* results.

## 2 Materials and methods

### 2.1 Animals

Eight clinically healthy crossbred pigs (*Sus scrofa domesticus*), aged 6–18 months old, were used as blood donors for *in vitro* experiments. Animals were housed at the Experiment Station of Istituto Zooprofilattico Sperimentale (IZS) of Sardinia (“Surigheddu”, Sassari, Italy) and were routinely monitored by trained veterinarians. Pigs were screened for ASFV, porcine circovirus 2 (PCV2), porcine parvovirus (PPV), porcine reproductive and respiratory syndrome virus (PRRSV), and *Mycoplasma hyopneumoniae*, to confirm their negative status, as previously described (21). Animal handling and experimental procedures (bleeding) were authorized by the Ministry of Health (Ref. 1232/2020-PR) and were carried out according to the Italian Legislative Decree No. 26 dated 4 March 2014 and in agreement with the Guide for the Use of Laboratory Animals issued by the Italian Ministry of Health (authorization no. 1232/2020-PR).

### 2.2 Generation of porcine monocyte-derived macrophages in vitro and stimulation

Monocyte-derived macrophages (moMΦ) were obtained from blood leukocytes using Petri dishes *in vitro*. In detail, cells were cultivated in media [RPMI-1640 with 10% fetal bovine serum (FBS), 100 U/mL penicillin, and 100 μg/mL streptomycin] [complete RPMI (cRPMI)] supplemented with 50 ng/mL of recombinant human M-CSF (hM-CSF) (Thermo Fisher Scientific, Waltham, MA, USA), as previously described (14, 15, 22). After 7 days in culture, moMΦ were detached, counted, and seeded in 12-well plates (10^6^ live cells per well) or 4-well chamber slides (Nunc Lab-Tek chamber slide system, Thermo Fisher Scientific) (3 × 10^5^ live cells per well) (14).

Twenty-four hours post-seeding, cells were left untreated (moMФ), or they were stimulated with scalar doses (20, 40, 100, and 200 ng/mL) of recombinant porcine IL-1β or IL-1Ra (both R&D Systems, Minneapolis, MN,USA). After 24-h morphology, expression of surface markers and cytokines was determined as described in Sections 2.3, 2.4, and 2.5, respectively.

For gene expression experiments (see Section 2.6) and the ability to respond to a TLR-3 ligand (see Section 2.7), cells were stimulated with a single dose of recombinant porcine IL-1β or IL-1Ra (100 ng/mL).

According to the manufacturer’s instructions, for both recombinant cytokines, the endotoxin level was below 0.0224 EU per 1 μg of the protein by the LAL method, which correspond to 0.00448 ng of LPS in 1 μg of products. The concentration used in the work (100–200 ng/mL of recombinant cytokines) corresponds to an extremely low dose of LPS (0.5–1 pg/mL), which does not trigger noticeable macrophage activation (23). In agreement, we did not detect cytokine release in response to 1 pg/mL of LPS (**Supplementary Figure 1**). In addition, 0.5 pg/mL of LPS did not prime the expression of pro-inflammatory mediators in macrophages upon a subsequent stimulation with polyinosinic–polycytidylic acid (poly I:C) (**Supplementary Figure 2**).

### 2.3 Microscopy

Cell morphology was investigated on macrophage subsets seeded in four-well chamber slides by phase contrast microscopy. Twenty-four hours post-stimulation with scalar doses of recombinant porcine IL-1β or IL-1Ra, cells were fixed with 4% paraformaldehyde and washed with phosphate-buffered saline (PBS), and phase contrast images were acquired using an inverted microscope (Olympus IX70, Segrate, Italy) equipped with a 20×/0.40 numeric aperture objective lens (15).

### 2.4 Flow cytometry

Flow cytometry was performed on macrophage subsets seeded in 12-well plates, with the aim of evaluating the expression of four cell surface markers, cellular dimension, and granularity, as we previously described (15). In details, 24 h post-stimulation, cells were harvested using 10 mM EDTA in PBS and were transferred to 5-mL round-bottom tubes (Corning, Corning, NY, USA). First, cells were stained with Zombie Aqua viability dye (BioLegend, San Diego, CA, USA) for 30 min at room temperature (RT) and were washed with PBS supplemented with 0.5% bovine serum albumin (BSA). Cells were subsequently stained with murine monoclonal antibodies: anti-porcine CD16-PE (clone G7, Thermo Scientific Pierce, Rockford, IL, USA), anti-human CD14-PerCP-Cy5.5 (clone Tuk4, Miltenyi Biotec, Bergisch Gladbach, Germany), anti-pig SLA class I (clone JM1E3, Bio-Rad Antibodies), and anti-pig SLA class II DR (clone 2E9/13, Bio-Rad Antibodies). Monoclonal antibodies anti‐human CD14 (Tuk4) cross‐react with pig (24). Expression of SLA class I and SLA class II DR was visualized by subsequent staining with secondary antibodies: BV421 rat anti-mouse IgG1 (clone A85-1, BD Horizon BD Biosciences, Franklin Lakes, NJ, USA) for SLA class I and BV786 rat anti-mouse IgG2b (clone R12-3, BD Horizon BD Biosciences) for SLA class II. Details of primary and secondary antibodies are provided in **Supplementary Table 1**, and all incubations were carried out for 15 min at 4 °C. After washing with PBS supplemented with 2% FBS, moMΦ were re-suspended in PBS supplemented with 2 mM EDTA and were analyzed with a FACS Celesta (BD Biosciences), acquiring 5000 live moMΦ. Data analysis was performed using BD FACS Diva Software (BD Biosciences): first doublets were excluded, then moMΦ were gated according to FSC and SSC values, dead cells were excluded according to viability staining, and finally staining for surface markers was assessed, as previously described (25, 26). Gates for surface markers were set using the corresponding unstained/isotype controls: IgG1 isotype control (Bio-Rad Antibodies) for SLA class I, IgG2b isotype control (Bio-Rad Antibodies) for SLA class II DR, mouse PE-IgG1 isotype control for CD16 (ZX3, Thermo Scientific Pierce), and unstained control for CD14, as we described in our previous work (22, 25, 26).

The mean fluorescence intensity (MFI) (geometric mean) of FSC-A and SSC-A of live macrophages was recorded, then the values of treated cells were expressed as fold change relative to the untreated control (moMΦ). For each marker, both percentage of positive cells and MFI values of positive cells (geometric mean) were calculated. For each marker, MFI values of the unstained control (geometric means) were near zero (**Supplementary Figure 3**).

### 2.5 Cytokine release by macrophages

MoMΦ were left untreated or polarized *in vitro* with diverse molecules. After 24 h, culture supernatants were collected, transferred into 15-mL Falcon tubes, centrifuged at 2,000×*g* for 3 min to remove cell debris, and stored at −80°C until analyzed. Levels of IL-1α, IL-1β, IL-1Ra, IL-6, IL-10, IL-12, and IL-18 were determined using Porcine Cytokine/Chemokine Magnetic Bead Panel Multiplex assay (Merck Millipore, Darmstadt, Germany) and a Bioplex MAGPIX Multiplex Reader (Bio‐Rad, Hercules, CA, USA) according to the manufacturer’s instructions, as previously described (15). The detection limits of IL-1α, IL-1β, IL-1Ra, IL-6, IL-10, IL-12, and IL-18 were 0.005, 0.122, 0.024, 0.024, 0.024, 0.024, and 0.024 ng/mL, respectively. Nevertheless, the standard curve allows extrapolation of values below the detection limit of the kit for IL-1α, IL-1β, IL-1Ra, IL-6, IL-10, IL-12, and IL-18. The lower extracted values were 0.003 (IL-1α), 0.006 (IL-1β), 0.010 (IL-1Ra), 0.009 (IL-6), 0.010 (IL-10), 0.005 (IL-12), and 0.005 (IL-18) ng/mL. Below these values, it was not possible to determine the concentrations, and their values were considered as zero.

### 2.6 Gene expression analysis

For each animal, three macrophage subsets were obtained: moMΦ, moM(IL-1β), and moM(IL-1Ra). RNA was extracted from the cell monolayers at 3 and 24 h post-stimulation, using an RNeasy Mini Kit (QIAGEN), as previously described (27). Genomic DNA was digested using an RNase-Free DNase set (QIAGEN). RNA concentration and purity were assessed using a NanoDrop 2000 spectrophotometer (Thermo Fisher). Total RNA (50 ng) was reverse-transcribed into cDNA using the RT² First Strand Kit (QIAGEN). RNA quality, genomic DNA contamination, and reverse-transcription efficiency were assessed using the RT² Profiler RNA QC PCR Array (QIAGEN). Real-time PCR was performed using the RT² Profiler PCR Array for pig antiviral genes (QIAGEN, Cat. No. PASS-122Z) on a CFX96TM Real-Time System (Bio-Rad, Milan, Italy). The list of genes included in the array is reported in **Supplementary Table 2**. Data were normalized using the reference genes included in the array (*B2M*, *GAPDH*, and *HPRT1*). Relative gene expression levels were calculated using the comparative Ct method, also known as the 2^−ΔΔCt^ method (28), with CFX Maestro software (ver. 4.1 Bio-Rad, Hercules, CA, USA) using the corresponding untreated condition (moMΦ) as control. Data were also *z*-score normalized for heatmap visualization using GraphPad Prism 10.10 (**Supplementary Table 3**).

### 2.7 Response to stimulation with a TLR-3 ligand

MoMФ were left untreated or stimulated with IL-1β (100 ng/mL) or IL-1Ra (100 ng/mL) (both R&D Systems), as described in Section 2.2. After 24 h, the culture media was removed and replaced with unsupplemented cRPMI or cRPMI supplemented with a TLR-3 agonist (poly I:C; Sigma-Aldrich) at 100 ng/mL. Then, 24 h post-stimulation, culture supernatants were removed, centrifuged at 2,000×*g* for 3 min (to remove cell debris), and stored at −80°C until determination of cytokine levels, as described in Section 2.5.

### 2.8 Statistical analysis

*In vitro* experiments were performed in technical duplicate and then the mean of each pair of technical duplicates was calculated. Each experiment was repeated using four diverse blood donor pigs (biological replicates). For both flow cytometry and ELISA data, the median and interquartile range (IQR) were used to provide descriptive statistics. Data were analyzed with the non-parametric Friedman test followed by a Dunn’s multiple comparison test. A *p*-value of less than 0.05 was considered statistically significant; *p* < 0.05 (*), *p* < 0.01 (**), and *p* < 0.001 (***).

PCR array for antiviral genes was instead analyzed using CFX Maestro software (ver. 4.1 Bio-Rad, Hercules, CA, USA). For each animal, three conditions were obtained: untreated (moMФ), moM(IL-β), and moM(IL-1Ra). For both IL-1β and IL-1Ra conditions, fold change in gene expression relative to the untreated (moMФ) control was calculated. The *p*-values were calculated based on a Student’s *t*-test of the replicate 2(^−ΔCt^) values for each gene in the untreated, IL-1β-treated, and IL-1Ra-treated groups; statistically significant difference was set as *p* < 0.05. GraphPad Prism 10.01 (GraphPad Software Inc., La Jolla, CA, USA) was used to graphically analyze the data, which were presented as a heatmap.

## 3 Results

### 3.1 Impact of IL-1β or IL-1Ra on porcine moMФ phenotype

**Figure 1:**
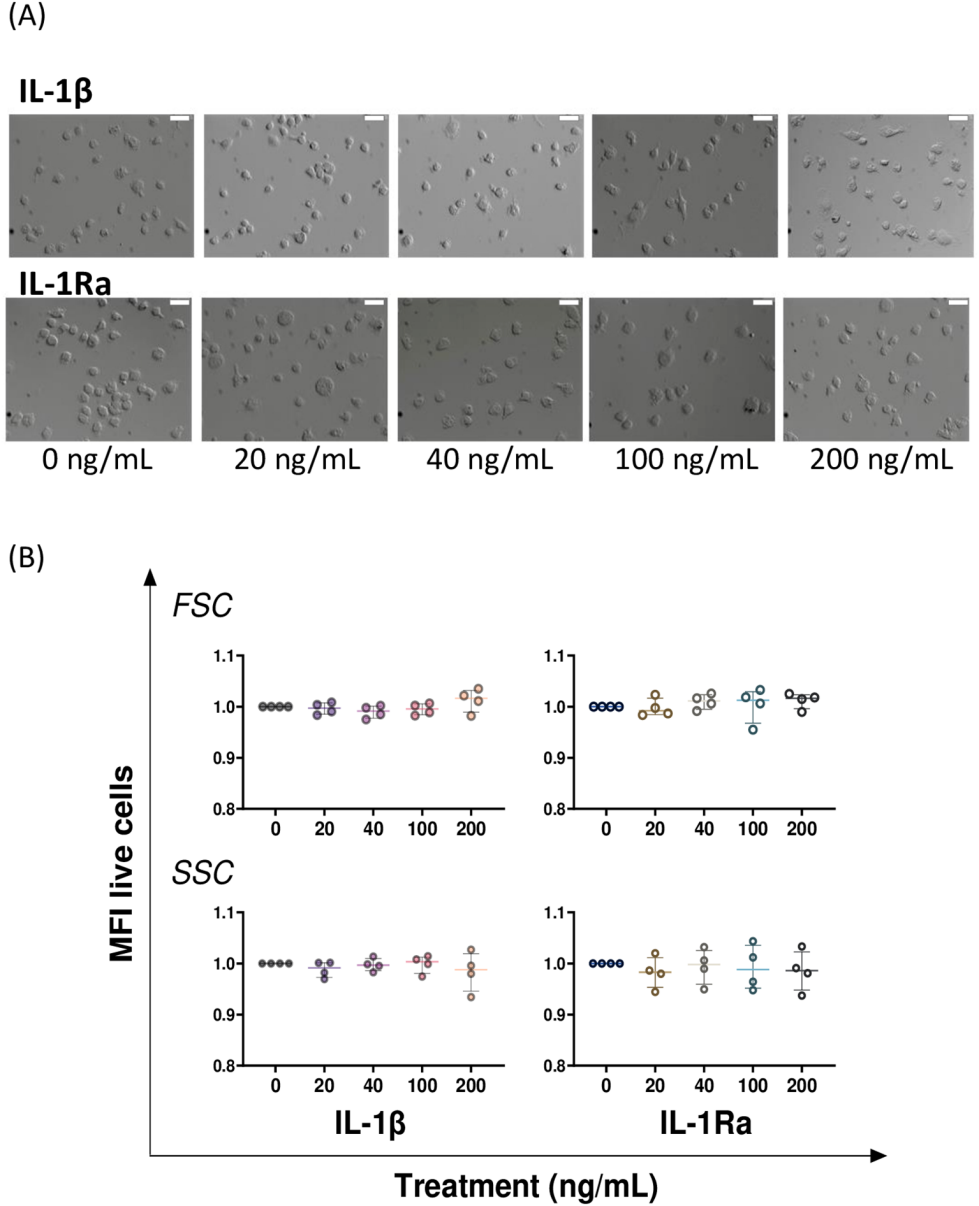
Impact of IL-1β and IL-1Ra on porcine moMФ morphology. Porcine moMΦ were left untreated, or they were stimulated with scalar doses of IL-1β (20, 40, 100, and 200 ng/mL) or IL-1Ra (20, 40, 100, and 200 ng/mL). After 24 h, morphologies were evaluated using phase contrast microscopy and flow cytometry: **(A)** Phase contrast microscopy images were acquired using an inverted microscope, with a magnification of 20×. Scale bar, 50 μm. Representative images are displayed. **(B)** Flow cytometry was employed to evaluate changes in moMΦ dimension [forward scatter (FSC)] and granularity [side scatter (SSC)]. Values of geometric mean of FSC-A and SSC-A in live macrophages were recorded and are presented as fold change relative to the untreated control (moMΦ). Data of four independent experiments using different blood donor pigs are presented; values of treated macrophages were compared to the untreated control (moMΦ), using a Friedman test followed by a Dunn’s multiple comparison test.

Porcine moMΦ were left untreated or stimulated with scalar doses of IL-1β or IL-1Ra. For both cytokines, the lower tested concentration was 20 ng/mL, in line with the concentration used for other cytokines in similar studies (14, 15), scaling up to 40, 100, and 200 ng/mL. After 24h, phenotypes of macrophage subsets were investigated with microscopy and flow cytometry. As expected, porcine moMΦ presented with a spherical shape with short “hairy” protrusions on their surface, in agreement with our previously published work (15). Both phase contrast microscopy and flow cytometry revealed that both IL-1β and its receptor antagonist (IL-1Ra) did not alter the morphology of these cells (**Figure 1**).

**Figure 2:**
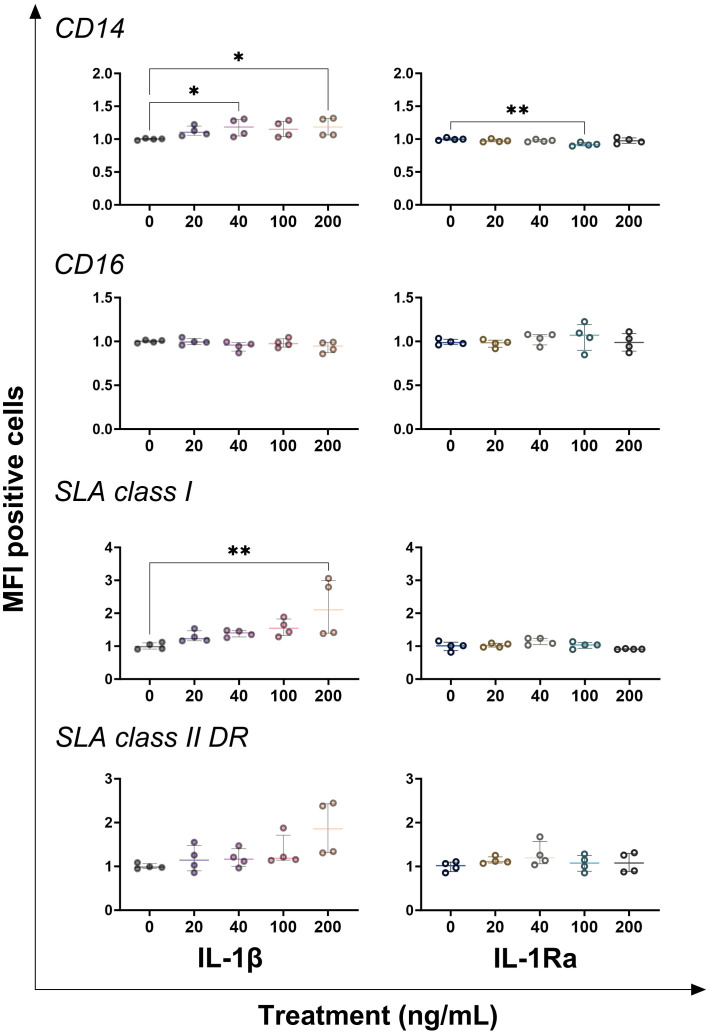
Impact of IL-1β and IL-1Ra on porcine moMФ surface marker expressions (mean fluorescence intensity). Porcine moMΦ were left untreated, or they were stimulated with scalar doses of IL-1β (20, 40, 100, and 200 ng/mL) or IL-1Ra (20, 40, 100, and 200 ng/mL). After 24 h, surface expression of CD14, CD16, SLA class II DR, and SLA class I was assessed by flow cytometry. For each marker, mean fluorescence intensity (MFI) values of positive cells was evaluated, and MFI data are presented as fold change relative to the untreated control (moMΦ). Data of four independent experiments using different blood donor pigs. Values of treated macrophages were compared to the untreated control (moMΦ), using a Friedman test followed by a Dunn’s multiple comparison test; *p < 0.05, **p < 0.01.

**Figure 3:**
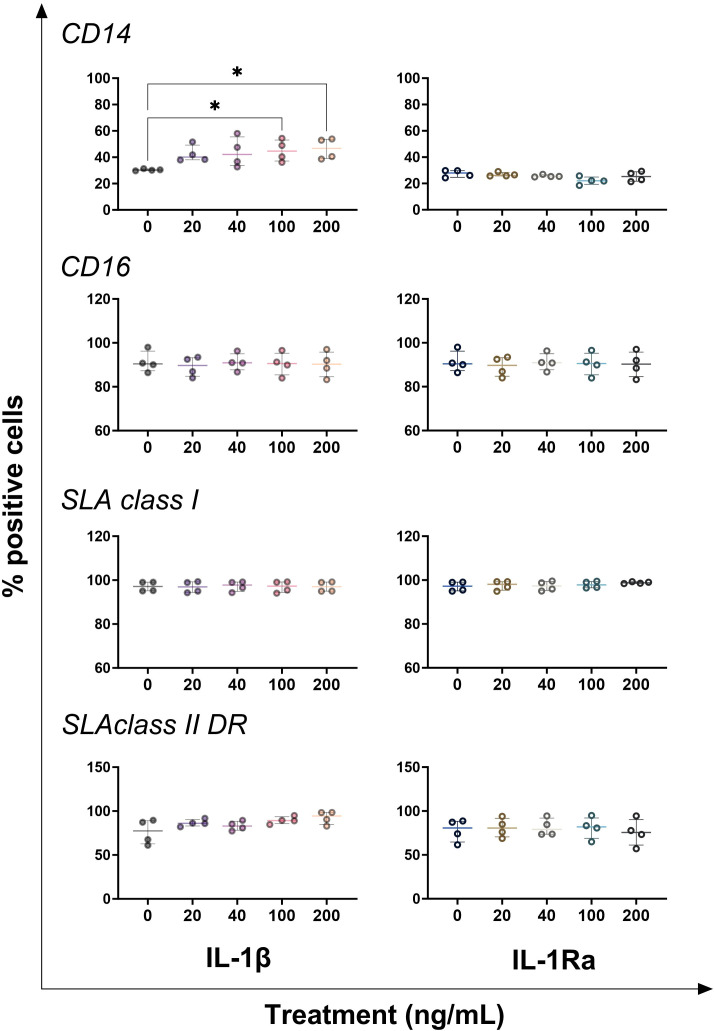
Impact of IL-1β and IL-1Ra on porcine moMФ surface marker expressions (% of positive cells). Porcine moMΦ were left untreated, or they were stimulated with scalar doses of IL-1β (20, 40, 100, and 200 ng/mL) or IL-1Ra (20, 40, 100, and 200 ng/mL). After 24 h, surface expression of CD14, CD16, SLA class II DR, and SLA class I was assessed by flow cytometry. For each marker, percentages of positive cells are shown. Data from four independent experiments using different blood donor pigs are presented. Values of treated macrophages were compared to the untreated control (moMΦ), using one-way ANOVA followed by a Friedman test and then by a Dunn’s multiple comparison test; *p < 0.05.

Then, expression of surface markers CD14, CD16, SLA class II, and SLA class I on porcine moMФ was investigated by flow cytometry, using previously published protocols (15). Stimulation with IL-1β resulted in upregulation of CD14, SLA class I, and SLA class II DR in a dose-dependent manner. In detail, stimulation with IL-1β enhanced CD14 levels significantly, in terms of both percentages of positive cells (200 ng/mL) and MFI of positive cells (at 40 and 200 ng/mL) (**Figures 2**, **3**). Stimulation with this interleukin triggered an increase of both SLA class I and II DR only in terms of MFI, but not percentages of positive cells, with significance at 200 ng/mL for SLA class I and a tendency for SLA class II (*p* = 0.056) (**Figures 2**, **3**). This cytokine did not alter the expression of CD16 (**Figures 2**, **3**). Stimulation with IL-1Ra did not significantly modulate the expression of these four markers, with the exception of a slight but statistically significant downregulation of CD14 at 100 ng/mL, only in terms of MFI and not percentages of positive cells (**Figures 2**, **3**).

### 3.2 Cytokine release by porcine moMФ in response to IL-1β or IL-1Ra stimulation

**Figure 4:**
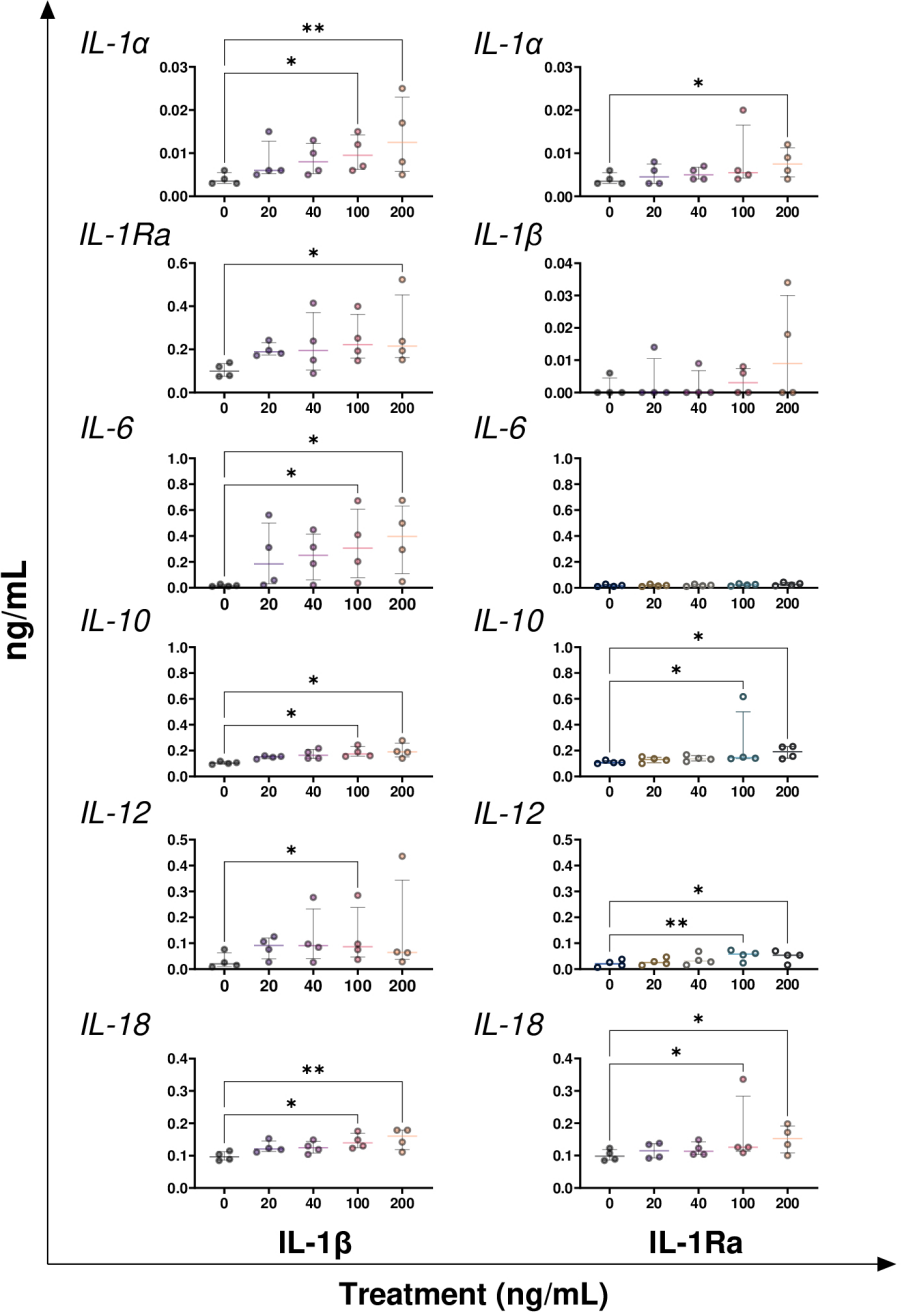
Release of key immune cytokines by moMФ following stimulation with IL-1β and IL-1Ra. Porcine moMΦ were left untreated, or they were stimulated with scalar doses of IL-1β (20, 40, 100, and 200 ng/mL) or IL-1Ra (20, 40, 100, and 200 ng/mL). After 24 h, levels of IL-1α, IL-1β, IL-1Ra, IL-6, IL-10, IL-12, and IL-18 in culture supernatants were determined by multiplex ELISA. Data of four independent experiments using different blood donor pigs are presented. Values of treated macrophages were compared to the untreated control (moMΦ), using a Friedman test followed by a Dunn’s multiple comparison test; *p < 0.05, **p < 0.01.

We next characterized the cytokine responses of porcine moMФ to IL-1β or IL-1Ra stimulation. Multiplex ELISA was employed to quantify the levels of the pro-inflammatory cytokines IL-6 and IL-12 in culture supernatants. As displayed in **Figure 4**, stimulation with IL-1β resulted in an enhanced release of IL-6 and IL-12, in a dose-dependent manner, with statistical significance at high concentration (100 and 200 ng/mL for IL-6; 100 ng/mL for IL-12). Stimulation with IL-1Ra did not modulate release of IL-6, but a significant release of IL-12 was detected at high concentration of IL-1Ra (100 and 200 ng/mL). Values of IL-6 in culture supernatants of IL-1Ra-treated samples were far lower than those in IL-1β-treated samples and always lower than 0.1 ng/mL (**Figure 4**).

Then, levels of the anti-inflammatory IL-10 in culture supernatants were assessed. As displayed in **Figure 3**, stimulation with either IL-1β or its receptor antagonist resulted in an enhanced release of IL-10 in a dose-dependent manner. For both IL-1β and IL-1Ra, significance was observed at concentration (100 and 200 ng/mL) (**Figure 4**).

Levels of four cytokines belonging to the IL-1 family (IL-1α, IL-1β, IL-1Ra, and IL-18) were next investigated. We observed that both recombinant porcine cytokines triggered a modest release of IL-1α, significant at high doses (100 and 200 ng/mL of IL-1β; 200 ng/mL of IL-1Ra). Stimulation with both IL-1β and IL-1Ra triggered a modest IL-18 release, with significance at high concentration (100 and 200 ng/mL) (**Figure 4**). Finally, our data revealed that stimulation with IL-1β triggered the release of its receptor antagonist by porcine moMФ in a dose-dependent manner, with significance at high doses (200 ng/mL). In contrast, stimulation with IL-1Ra did not trigger the release of IL-1β by porcine moMФ (**Figure 4**).

### 3.3 Impact of IL-1β or IL-1Ra on the ability of porcine moMФ to respond to a TLR-3 ligand

We next evaluated the ability of moM(IL-1β) or moM(IL-1Ra) to respond to stimulation with poly I:C, which is a TLR-3 ligand. We selected a concentration of 100 ng/mL for both IL-1β and L-1Ra, considering that this dose had a significant impact on macrophage functionality for both IL-1 family members.

**Figure 5:**
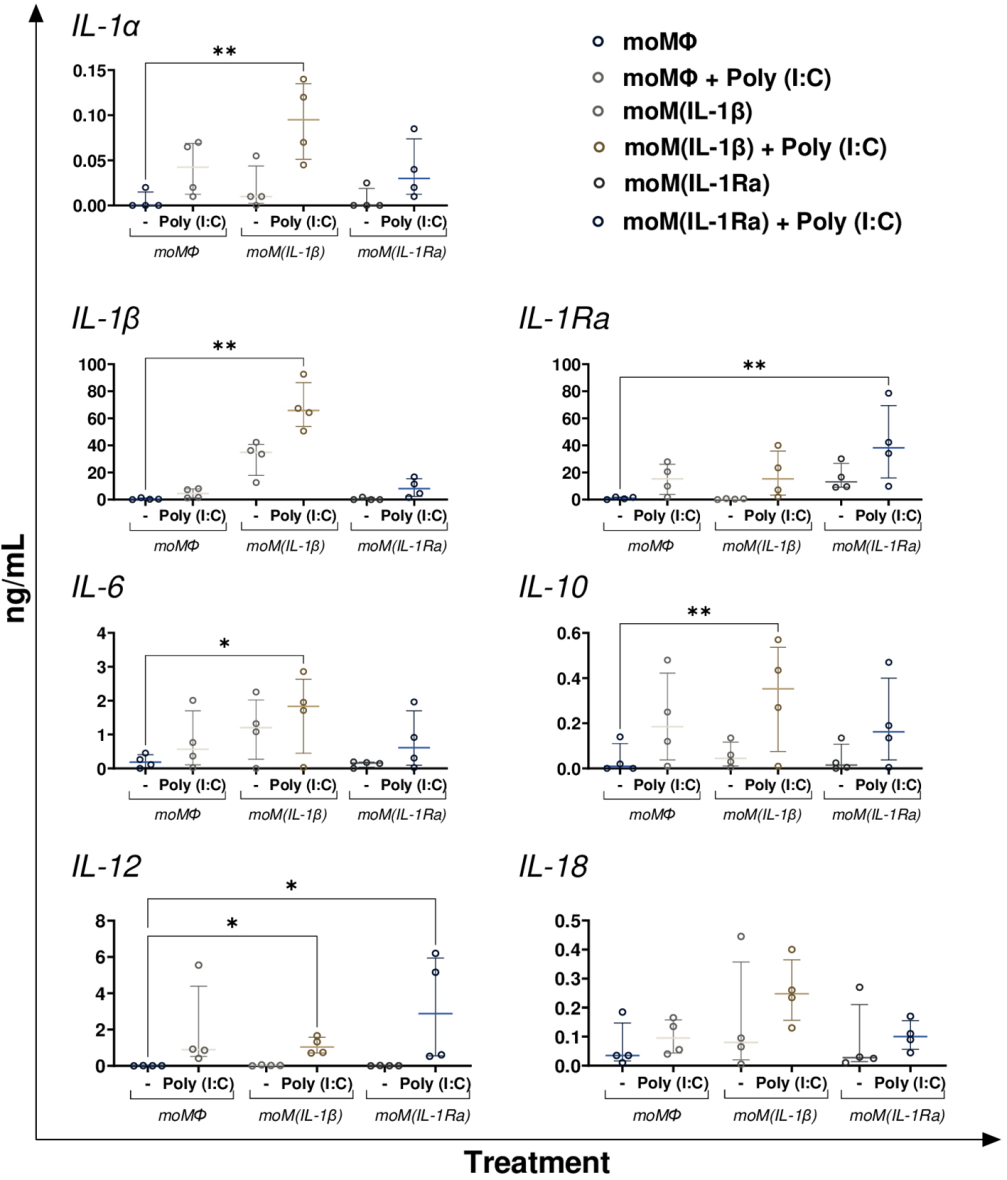
Ability of macrophage subsets to release key immune cytokines in response to TLR-3 agonist stimulation. The moMΦ were left untreated, or they were stimulated with IL-1β (100 ng/mL) or IL-1Ra (100 ng/mL). Then, 24 h later, culture supernatants were replaced with fresh media and cells were left untreated or activated using a TLR-3 ligand (poly I:C, 100 ng/mL); 24 h later, the amounts of IL-1α, IL-1β, IL-1Ra, IL-6, IL-10, IL-12, and IL-18 in culture supernatants were determined using a multiplex ELISA. Data from four independent experiments utilizing different blood donors are presented. Values of treated macrophages were compared to the untreated control (moMΦ), using a Friedman test followed by a Dunn’s multiple comparison test; *p < 0.05, **p < 0.01.

Levels of IL-1 family members (IL-1α, IL-1β, IL-1Ra, and IL-18) and other key immune cytokines (IL-6, IL-10, and IL-12) in culture supernatants of macrophage subsets untreated or stimulated with poly I:C are presented in **Figure 5**. Both moM(IL-1β) and moM(IL-1Ra) released IL-12 in response to poly I:C stimulation, whereas only moM(IL-1β) released IL-1α, IL-1β, IL-6, and IL-10 in response to poly I:C stimulation with statistical significance (**Figure 5**). MoM(IL-1Ra) released IL-1Ra in response to this TLR-3 ligand, and no significant differences between poly I:C-stimulated and the unstimulated condition was observed for IL-18 (**Figure 5**).

### 3.4 Induction of antiviral gene expression by porcine moMФ in response to IL-1β or IL-1Ra stimulation

Finally, we characterized the expression of antiviral genes in porcine moMФ stimulated with IL-1β or IL-1Ra (both at 100 ng/mL). Two time points were selected: 3 h post-stimulation (early time point) and 24 h post-stimulation (in parallel to measurement of cytokine release and surface marker expression).

**Figure 6:**
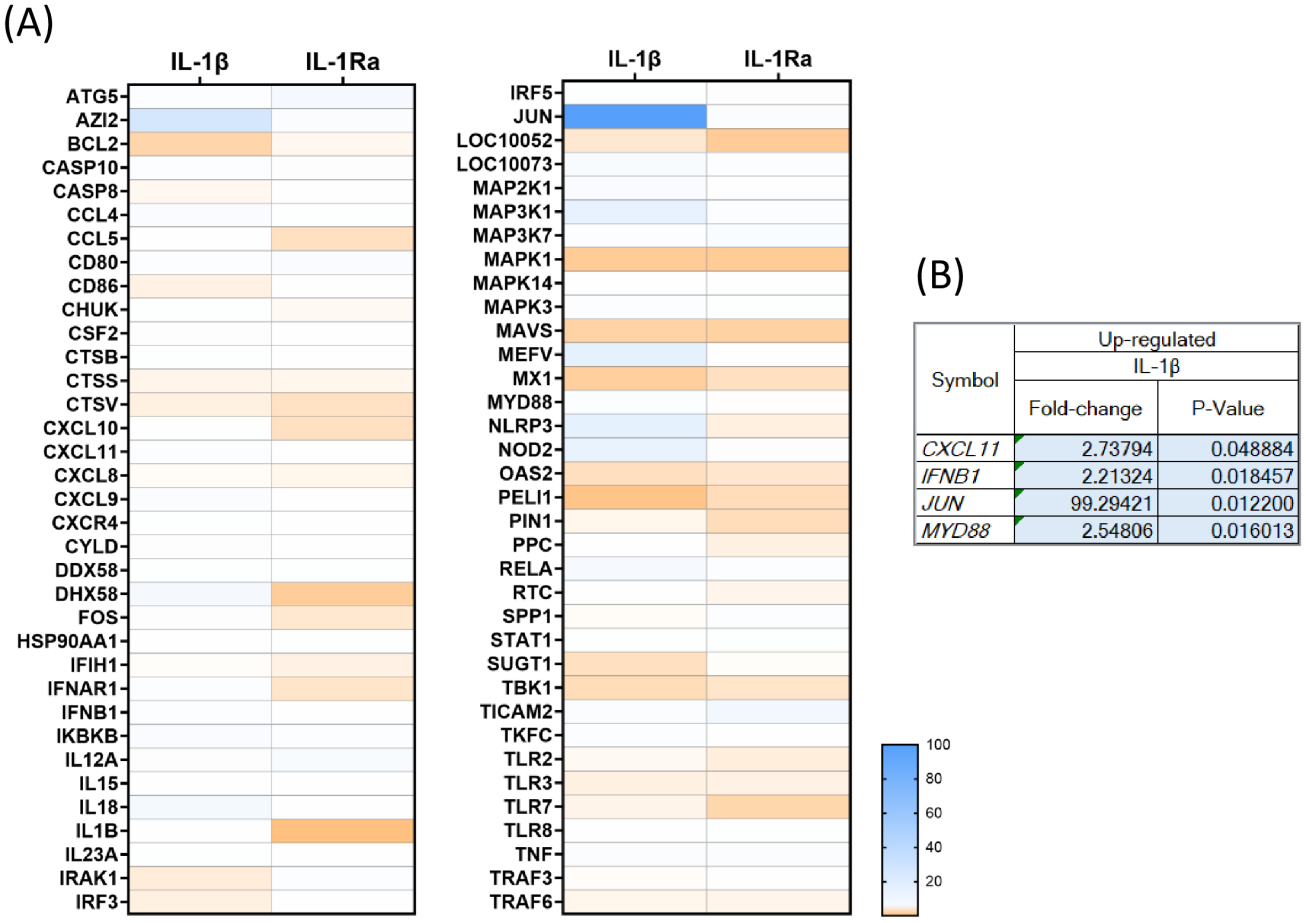
Modulation of 70 antiviral genes in porcine moMΦ 3 h post-stimulation with IL-1β and IL-1Ra. Porcine moMΦ were left untreated, or they were stimulated with IL-1β (100 ng/mL) or IL-1Ra (100 ng/mL). At 3 h post-stimulation, macrophages were analyzed using the RT2 Profiler PCR Array for 70 antiviral genes. **(A)** The heatmap illustrates fold change expression of immune-related genes. Data of four independent experiments utilizing different blood donors are presented. For each cytokine, fold change in gene expression relative to the untreated control (moMФ) was calculated, and colors represent these fold changes: blue represents the highest value, white denotes the baseline value (fold change = 1), and orange indicates the smallest value. **(B)** Statistically significantly upregulated (fold change > 2, *p*-value < 0.05) and downregulated (fold change < 0.5, *p*-value < 0.05) genes are presented, with the corresponding fold change and *p*-value.

**Figure 7:**
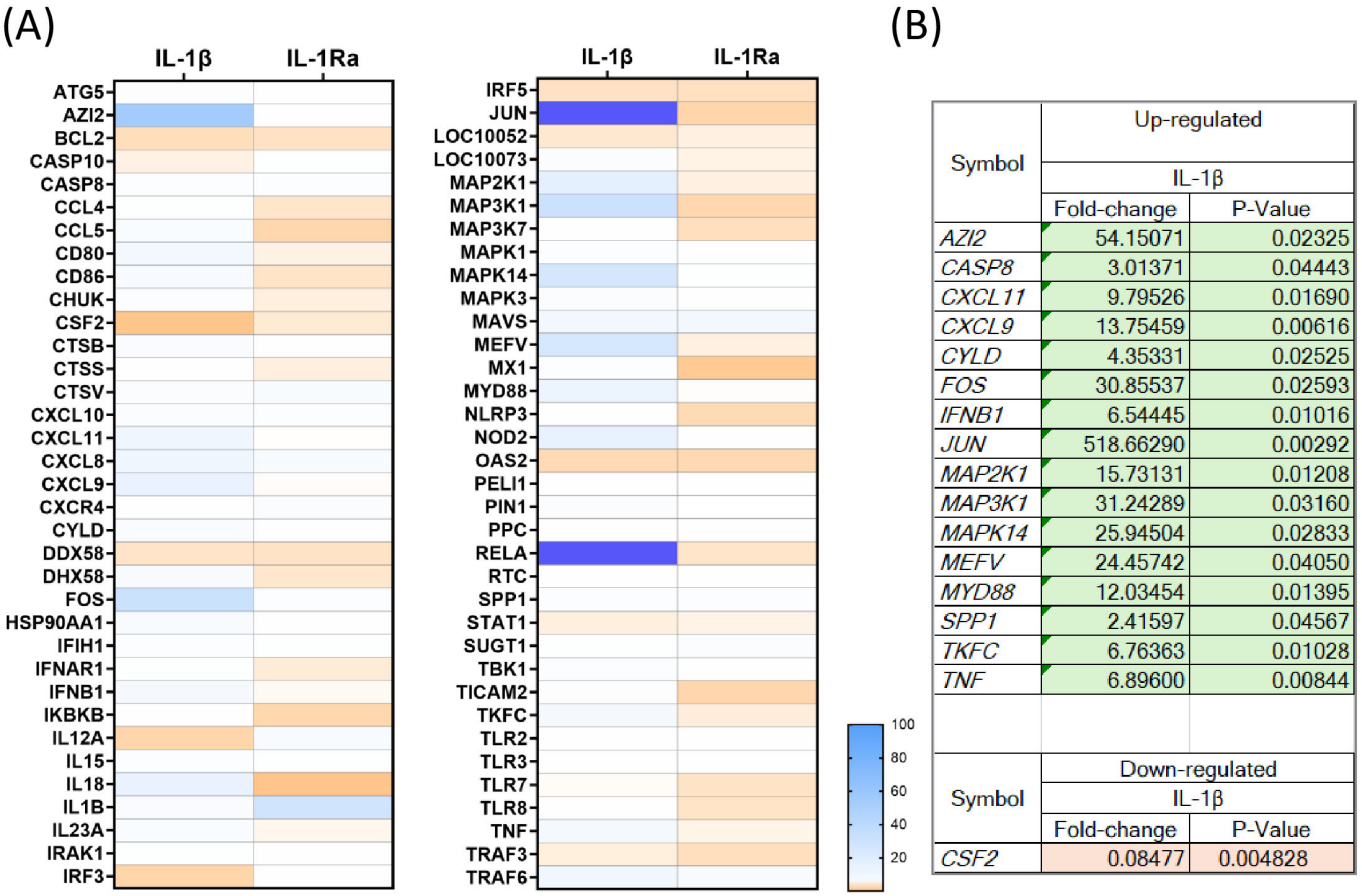
Modulation of 70 antiviral genes in porcine moMΦ 24 h post-stimulation with IL-1β and IL-1Ra. Porcine moMΦ were left untreated, or they were stimulated with IL-1β (100 ng/mL) or IL-1Ra (100 ng/mL). At 24 h post-stimulation, macrophages were analyzed using the RT2 Profiler PCR Array for 70 antiviral genes. **(A)** The heatmap illustrates fold change expression of immune-related genes. Data from four independent experiments utilizing different blood donors are presented. For each cytokine, fold change in gene expression relative to the untreated control (moMФ) was calculated, and colors represent these fold changes: blue represents the highest value, white denotes the baseline value (fold change = 1), and orange indicates the smallest value. **(B)** Statistically significantly upregulated (fold change > 2, *p*-value < 0.05) and downregulated (fold change < 0.5, *p*-value < 0.05) genes are presented, with the corresponding fold change and *p*-value.

For each time point, the gene expression of moM(IL-1β) or moM(IL-1Ra) was first normalized to the untreated control (moMФ), and the upregulated (blue) and downregulated (orange) genes are displayed (**Figures 6**, **7**). For each gene, the fold change normalized to the untreated control and the corresponding *p*-value are reported in **Supplementary Table 2**. Fold changes > 2.0 and < 0.5, with *p*-value < 0.05, were regarded as significant variations (**Figures 6**, **7**). In addition, *z*-score normalized data are presented in **Supplementary Figure 4**, **S5**.

At 3 h, our data revealed that stimulation with IL-1β (100 ng/mL) triggered significant upregulation of few genes coding for molecules with pro-inflammatory and antiviral activities. In particular, we observed a strong upregulation of *JUN*, with a fold change of 99.924. A significant increase was also observed for *IFNB1*, *CXCL11*, and the adaptor protein *MYD88*, but with low intensity (fold change < 3) (**Figure 6**). A tendency (*p* < 0.1) was also detected for the chemokine *CXCL9* (fold change = 3.793) and the mitogen-activated protein kinases *MAP2K1* (fold change = 4.223) and *MAP3K1* (fold change = 14.062) (**Supplementary Table 2**). No significant upregulation was observed in cells stimulated with IL-1Ra. At 3 h, for both IL-1 and its receptor antagonist, no genes were downregulated with fold change < 0.5 and *p*-value < 0.05 (**Figure 6**, **Supplementary Table 2**).

At 24 h, we observed that stimulation with IL-1β (100 ng/mL) resulted in significant upregulation of several genes coding for molecules with pro-inflammatory and antiviral activities. In particular, we observed a strong upregulation of *JUN*, with fold change > 500. At this time point, we observed a strong upregulation for *AZI2* and *FOS*, with fold changes of 54.151 and 30.855, respectively. Three mitogen-activated protein kinases were strongly upregulated: *MAP2K1, MAP3K1*, and *MAPK14*, all with a fold change higher than 20. A significant increase was also observed for *IFNB1*, *CXCL11*, and the adaptor protein *MYD88*, with fold changes higher than those detected at 3 h post-stimulation (6.544, 9.795, and 12.035, respectively). Stimulation with IL-1β resulted in increased expression of two genes encoding for pro-inflammatory cytokines (*TNF* and *CXCL9*) and the receptor for pyrin (*MEFV*), which is an important modulator of inflammation. Finally, a significant increase was observed for *CASP8, CYLD, SPP1*, and *TKFC* (**Figure 7**). In contrast, no genes were significantly upregulated following stimulation with IL-1Ra, but a tendency was observed for *IL1B*, with a fold change of 27.936 and a *p*-value = 0.076 (**Figure 7** and **Supplementary Table 2**). At this time point, for both IL-1 and its receptor antagonist, no genes were downregulated with fold change < 0.5 and *p*-value < 0.05, with the exception of a downregulation of *CSF2* in response to IL-1β stimulation (**Figure 7**; **Supplementary Table 2**).

## 4 Discussion

Pigs are attracting increasing attention as a close-to-human biomedical model due to their physiological and immunological similarities to humans (29). Nevertheless, the pig immune system has not been fully portrayed yet, also at the level of macrophage polarization. We previously described that porcine M1 possesses a phenotype similar to those of humans and mice, characterized by increased expression of SLA-I and SLA-II, and the release of pro-inflammatory cytokines, although they were associated with high expression of *IL-1Ra* and *IL-33*, which are two cytokines commonly associated with an M2 polarization in humans and mice (15, 30). In addition, we observed that porcine macrophages exposed to diverse “M2-related” polarizing factors (IL-4, IL-10, TGF-β, and dexamethasone) did not express/release high levels of IL-10 (15). Despite these peculiarities, the impact of IL-1 family members on the porcine macrophage phenotype was poorly investigated so far. We therefore thoroughly analyzed the effect of two key IL-1 family members (the pro-inflammatory IL-1β and its receptor antagonist IL-1Ra) on macrophage polarization.

We observed that exposure to scalar doses of IL-1β resulted in upregulation of CD14, a co-receptor for the recognition of LPS by TLR4 (31), indicating an enhanced readiness to sense microbial products, although functional validation is required.

In line with these findings, we next observed that macrophages stimulated with high levels of this cytokine released high levels of pro-inflammatory cytokines, such as IL-1α, IL-6, IL-18, and IL-12. Similarly, previous studies in humans reported that moM(IL-1β) presented a pro-inflammatory phenotype, with increased expression of pro-inflammatory *IL1B, IL6*, and *CCL2* (32). Overall, in both humans and pigs, IL-1β promotes a polarization of macrophages toward a pro-inflammatory and anti-microbial phenotype.

We also observed that IL-1β triggered the release of the anti-inflammatory IL-10 and the receptor antagonist IL-1Ra by porcine macrophages, which, in human and mice, are mainly associated with an M2 polarization. Nevertheless, we and others have previously reported that, in pigs, both IL-10 and IL-1Ra are released mainly by M1 (14, 15, 33). It seems that, in pigs, macrophages simultaneously release both anti-inflammatory and pro-inflammatory cytokines, like an attempt to dampen inflammation once it started.

Our results revealed that IL-1Ra had instead a modest effect on untreated macrophages (moMΦ), with no modulation of any of the tested surface markers, with the exception of a small decrease of CD14 at 100 ng/mL. IL-1Ra is released to block IL-1α and IL-1β pro-inflammatory effects; thus, it is plausible that in the absence of these interleukins, its effects are modest. One possible explanation is that there is a small basal release of IL-1α and IL-1β from moMΦ, which is blocked at high doses of IL-1Ra, with subsequent downregulation of CD14.

IL-1Ra did not trigger the release of pro-inflammatory IL-1β and IL-6, but instead a small release of IL-1α and IL-18 was observed. Nevertheless, we previously observed that, in pigs, IL-18 release can be associated with an M2 phenotype (15). In addition, IL-1Ra induced IL-10 released by porcine moMΦ, in line with its anti-inflammatory action. This suggests that IL-1Ra has both an indirect role as a “blocker” of the IL-1 inflammatory signaling and a direct impact on macrophages.

Then, we investigated the ability of moM(IL-1β) and moM(IL-1Ra) to respond to a TLR-3 ligand (poly I:C). TLR-3 is an intracellular receptor able to sense viral dsRNA, with subsequent activation of signaling cascades, which culminate in the release of several pro-inflammatory cytokines (34, 35). As we previously observed (15), poly I:C stimulation resulted in the significant release of IL-12, IL-6, IL-1α, and IL-1β from moM(IL-1β). As expected, lower levels of the pro-inflammatory cytokines IL-1α, IL-1β, and IL-6 were observed in the culture supernatants of poly I:C-stimulated moM(IL-1Ra) compared to moM(IL-1β), although no differences were observed in terms of IL-12. We previously observed that porcine macrophages treated with IL-10 or other immunosuppressive molecules (dexamethasone) acquired an anti-inflammatory phenotype, with a lower ability to release IL-12 in response to poly I:C stimulation (15). In contrast, in this work, our data revealed that moM(IL-1Ra) were able to quickly release IL-12 in response to TLR-3 ligand stimulation, suggesting that this subset was still able to promptly respond to danger signal.

Finally, we analyzed the effect of these cytokines on the expression of 70 immunity genes.

Although a small number of animals were tested (four for each time point), these preliminary *in vitro* data showed that IL-1β is a strong pro-inflammatory cytokine, which triggered the upregulation of genes encoding for pro-inflammatory cytokines (*TNF*, *CXCL9*, and *CXCL11*), the adaptor protein *MYD88*, and *MEFV*, the latter a gene encoding for pyrin, involved in the assembly of inflammasome complexes (36).

We also observed that IL-1β triggered a strong upregulation of *JUN*, a gene coding for a transcription factor with a significant role in classical macrophage activation. Our results are in line with previous data observed in mice, where researchers described that *JUN* is required for full expression of *IL1B* and of additional genes involved in classical inflammation in macrophages treated with LPS and other immune-stimulatory molecules (37). A significant upregulation of the gene coding for another transcription factor (*FOS*) was observed, which also plays a role in promoting inflammation (38).

Stimulation with IL-1β resulted also in an early and strong upregulation of mitogen-activated protein kinases, particularly *MAP2K1* and *MAP3K1*, which were upregulated as soon as 3 h post-stimulation. In mammalian species, these proteins are involved in all aspects of immune responses, from the initiation phase of innate immunity, to activation of adaptive immunity, and to cell death when immune function is complete (39). In particular, *MAP3K1* regulates the production of interferons, inflammatory mediators, and other antiviral substances in response to viral infection, which are essential to the host’s defense against viral invasion (40). In agreement, a strong increase of the gene coding for the antiviral cytokines IFN-β was detected as early as 3 h post-stimulation.

Then, we observed modulation of genes involved in cell death. Stimulation with IL-1β resulted in enhanced expression of both *CASP8* and *AZI2*, which possess antithetic actions. *CASP8* encodes for the enzyme Caspase-8, which promotes apoptosis, and it is involved in other cell death processes, such as pyroptosis and necroptosis (41). Interestingly, IL-1β stimulation also resulted in a strong upregulation for *AZI2*, with fold change > 50, which encodes for an adapter protein, with an important role in protection against TNF-induced cell death (42). This is likely a host’s protective mechanism, to avoid massive cell death triggered by high levels of pro-inflammatory TNF. Future studies should investigate the impact of IL-1β and its receptor antagonist on diverse types of cell death, including apoptosis, pyroptosis, and necroptosis.

Although only a small number of animals were tested (four for each time point), our data revealed that no genes were significantly upregulated following stimulation with IL-1Ra, confirming its regulatory role. However, 24 h post-stimulation with IL-1Ra, we detected an increasing trend for *IL1B* expression. This seems to be another protective mechanism: the system tries to balance itself, this time to prevent excessive inhibition.

In this preliminary work, we investigated the expression of 70 immunity genes, but future studies are needed to provide a broader portrait of the impact of IL-1β and IL-1Ra on porcine macrophages. Previous work reported that three chemokine receptors (CCR1, CCR2, and CCR5) play a crucial role in the recruitment of monocytes and macrophages in inflammation (43). In addition, a recent study in mice showed that the expression of *CCR1*, *CCR2*, and *CCR5* in lung macrophages is specific and malleable in response to discrete inflammatory stimuli (44). Future studies should investigate the expression of these and other chemokine receptors in porcine macrophages in response to IL-1β and its receptor antagonist, also using other techniques like microarray analysis and RNA sequencing (RNA-seq). Transcriptomic analysis would provide a more comprehensive understanding of porcine macrophage responses to these stimuli.

Overall, our data showed that IL-1β polarizes porcine macrophages toward a pro-inflammatory phenotype, but a strong release of anti-inflammatory cytokines, including IL-1Ra, was also observed. In addition, IL-1Ra stimulation led to a small release of IL-1α and an increasing trend in *IL1B* expression. It seems that, in pigs, macrophages try to keep a balance between these two cytokines, as a defense mechanism to control inflammation. Overall, in this work, we provided a better understanding of porcine macrophage polarization, which could benefit translational studies using this large-animal model.

## Data Availability

The raw data supporting the conclusions of this article will be made available by the authors, without undue reservation.
