# Supplementary material for: Phenotypic and functional changes to porcine monocyte-derived macrophages triggered by IL-1β and its receptor antagonist IL-1Ra
Source: Front Immunol. 2026 Aug 3;17:1869718. doi: 10.3389/fimmu.2026.1869718 (PMC13477873; doi:10.3389/fimmu.2026.1869718)
Supplement: Supplementary file 1 [file DataSheet1.pdf]

Table S1. Antibodies used for flow cytometry.

| Antibody | Reactivity | Clone | Isotype | Conjugate | Concentration (mg/mL) | Workingdilution* |
|---|---|---|---|---|---|---|
| **Primary Antibodies** | | | | | | |
| SLA class I | Pig | JM1E3 | Mouse IgG1 | - | 0.1 | 1/40 |
| SLA class II DR | Pig | 2E9/13 | Mouse IgG2b | - | 0.1 | 1/25 |
| CD14 | Human** | Tuk4 | Mouse IgG2a | PerCP | ND | 1/5 |
| CD16 | Pig | G7 | Mouse IgG1 | PE | ND | 1/4 |
| **Secondary Antibodies** | | | | | | |
| Anti-IgG1 | Mouse | A85-1 | Rat IgG1 | BV421 | 0.2 | 1/25 |
| Anti-IgG2b | Mouse | R12-3 | Rat IgG2a | BV786 | 0.2 | 1/25 |

ND: notdetermined.*10 µL of dilutedantibodywereadded to cellpellets (100 µLtotal).
**Cross-reactive with pig [Jacobsen et al., 1993].

Table S2. Fold regulation of genes, compared to untreated control (moMΦ), and the corresponding p value at 3 and 24 h post-stimulation.

| | 3 h | | | | 24 h | | | |
|---|---|---|---|---|---|---|---|---|
| | IL-1β | | IL-1Ra | | IL-1β | | IL-1Ra | |
| Symbol | Fold-change | P-Value | Fold-change | P-Value | Fold-change | P-Value | Fold-change | P-Value |
| *ATG5* | 2,66425 | 0,449247 | 6,28601 | 0,503950 | 2,07783 | 0,379843 | 1,08257 | 0,883178 |
| *AZI2* | 25,79751 | 0,163654 | 3,25112 | 0,563892 | 54,15071 | 0,023250 | 1,05999 | 0,967070 |
| *BCL2* | 0,36931 | 0,604949 | 0,88415 | 0,959931 | 0,46327 | 0,517259 | 0,52608 | 0,563829 |
| *CASP10* | 2,85071 | 0,409113 | 2,12384 | 0,631661 | 0,78982 | 0,689390 | 1,91459 | 0,681463 |
| *CASP8* | 0,88113 | 0,714655 | 1,33829 | 0,869936 | 3,01371 | 0,044428 | 2,60666 | 0,303690 |
| *CCL4* | 4,24853 | 0,124086 | 1,77237 | 0,104093 | 1,76780 | 0,560957 | 0,58954 | 0,621671 |
| *CCL5* | 1,03599 | 0,973749 | 0,54203 | 0,679005 | 4,06613 | 0,096226 | 0,36462 | 0,699388 |
| *CD80* | 2,92086 | 0,478801 | 4,24835 | 0,346692 | 7,44650 | 0,348114 | 0,78946 | 0,842444 |
| *CD86* | 0,80181 | 0,912827 | 1,51912 | 0,816416 | 5,62252 | 0,453973 | 0,56141 | 0,773138 |
| *CHUK* | 1,85049 | 0,139148 | 0,90597 | 0,831365 | 2,97595 | 0,110705 | 0,74005 | 0,660716 |
| *CSF2* | 2,06284 | 0,574469 | 1,45111 | 0,870309 | 0,08477 | 0,004828 | 0,65358 | 0,697331 |
| *CTSB* | 1,97079 | 0,523396 | 1,07871 | 0,964447 | 4,36312 | 0,246467 | 1,21284 | 0,815127 |
| *CTSS* | 0,83732 | 0,494041 | 0,86486 | 0,760325 | 1,13310 | 0,721256 | 0,73832 | 0,598160 |
| *CTSV* | 0,77430 | 0,826992 | 0,55426 | 0,347722 | 3,76203 | 0,591121 | 4,53112 | 0,208421 |
| *CXCL10* | 1,26077 | 0,560683 | 0,53680 | 0,525350 | 3,65954 | 0,351353 | 3,05794 | 0,355431 |
| *CXCL11* | 2,73794 | 0,048884 | 1,26819 | 0,747902 | 9,79526 | 0,016899 | 0,97295 | 0,974959 |
| *CXCL8* | 0,92906 | 0,951743 | 0,85481 | 0,887815 | 10,28474 | 0,216966 | 5,10916 | 0,075126 |
| *CXCL9* | 3,79723 | 0,078810 | 1,35630 | 0,785507 | 13,75459 | 0,006162 | 0,94752 | 0,945362 |
| *CXCR4* | 1,78516 | 0,040491 | 1,16878 | 0,612617 | 1,49298 | 0,602191 | 3,69540 | 0,473453 |
| *CYLD* | 2,11674 | 0,113771 | 1,15556 | 0,773198 | 4,35331 | 0,025255 | 1,13623 | 0,769893 |
| *DDX58* | 2,01609 | 0,437405 | 1,89670 | 0,480759 | 0,57155 | 0,479691 | 0,54389 | 0,390703 |
| *DHX58* | 6,24774 | 0,217825 | 0,23146 | 0,678056 | 4,84698 | 0,289403 | 0,61335 | 0,769082 |
| *FOS* | 2,14052 | 0,293458 | 0,64446 | 0,735979 | 30,85537 | 0,025931 | 2,65361 | 0,124257 |
| *HSP90AA1* | 1,44770 | 0,881613 | 1,89985 | 0,733237 | 4,74614 | 0,290333 | 1,11918 | 0,899129 |
| *IFIH1* | 0,96145 | 0,983342 | 0,79067 | 0,924047 | 2,86522 | 0,379997 | 1,83213 | 0,727530 |
| *IFNAR1* | 2,26313 | 0,727048 | 0,59521 | 0,817590 | 1,76402 | 0,892373 | 0,69001 | 0,891986 |
| *IFNB1* | 2,21324 | 0,018457 | 1,18812 | 0,301713 | 6,54445 | 0,010159 | 0,90485 | 0,829675 |
| *IKBKB* | 4,35135 | 0,675238 | 2,35916 | 0,796127 | 1,56585 | 0,833040 | 0,37106 | 0,702378 |
| *IL12A* | 2,05592 | 0,694687 | 4,62026 | 0,256672 | 0,34642 | 0,212551 | 4,62177 | 0,129135 |
| *IL15* | 1,38149 | 0,494451 | 1,25688 | 0,781361 | 2,22505 | 0,230883 | 1,77018 | 0,568770 |
| *IL18* | 7,22478 | 0,350437 | 1,17515 | 0,942260 | 13,50582 | 0,487185 | 0,07923 | 0,342664 |
| *IL1B* | 1,13827 | 0,776623 | 0,05001 | 0,215481 | 4,18590 | 0,591900 | 27,93615 | 0,075996 |
| *IL23A* | 1,99437 | 0,190553 | 1,09294 | 0,832191 | 4,02562 | 0,171292 | 0,85567 | 0,850971 |
| *IRAK1* | 0,72067 | 0,770781 | 2,30029 | 0,431767 | 1,66795 | 0,518082 | 1,00419 | 0,997335 |
| *IRF3* | 0,77699 | 0,679973 | 1,17904 | 0,853793 | 0,33157 | 0,525364 | 1,25275 | 0,775386 |

| | | | | | | | | |
|---|---|---|---|---|---|---|---|---|
| *IRF5* | 1,66970 | 0,280046 | 2,13007 | 0,400637 | 0,53913 | 0,490004 | 0,51547 | 0,488576 |
| *JUN* | 99,29421 | 0,012200 | 3,24637 | 0,425114 | 518,66291 | 0,002917 | 0,33610 | 0,626685 |
| *LOC10052* | 0,65147 | 0,805783 | 0,21571 | 0,627034 | 0,62636 | 0,836156 | 0,75980 | 0,901267 |
| *LOC10073* | 4,51910 | 0,370919 | 2,72109 | 0,558777 | 3,08704 | 0,417186 | 0,80036 | 0,849666 |
| *MAP2K1* | 4,22272 | 0,088800 | 1,08685 | 0,927287 | 15,73131 | 0,012077 | 0,75763 | 0,726577 |
| *MAP3K1* | 14,06161 | 0,065635 | 2,54124 | 0,521288 | 31,24289 | 0,031601 | 0,37173 | 0,236011 |
| *MAP3K7* | 2,47859 | 0,530044 | 3,94567 | 0,149075 | 1,22650 | 0,879889 | 0,48803 | 0,601427 |
| *MAPK1* | 0,21842 | 0,129022 | 0,21475 | 0,497863 | 2,98824 | 0,100087 | 1,09834 | 0,938394 |
| *MAPK14* | 1,24482 | 0,864958 | 1,47258 | 0,747243 | 25,94504 | 0,028334 | 1,93201 | 0,752623 |
| *MAPK3* | 2,57939 | 0,253745 | 1,91943 | 0,273627 | 3,24462 | 0,211506 | 1,12142 | 0,744052 |
| *MAVS* | 0,33224 | 0,565353 | 0,30670 | 0,592012 | 8,17961 | 0,208775 | 7,51675 | 0,260468 |
| *MEFV* | 15,34682 | 0,133807 | 1,58134 | 0,595209 | 24,45742 | 0,040501 | 0,75424 | 0,859882 |
| *MX1* | 0,26934 | 0,587097 | 0,52183 | 0,212523 | 2,90595 | 0,059186 | 0,15015 | 0,361748 |
| *MYD88* | 2,54806 | 0,016013 | 0,98295 | 0,917161 | 12,03454 | 0,013951 | 0,96152 | 0,960850 |
| *NLRP3* | 15,22600 | 0,146261 | 0,76487 | 0,937882 | 1,19739 | 0,911163 | 0,41662 | 0,629718 |
| *NOD2* | 13,31886 | 0,249211 | 2,16421 | 0,688854 | 14,22544 | 0,277150 | 1,17139 | 0,942118 |
| *OAS2* | 0,51731 | 0,552742 | 0,62286 | 0,659293 | 0,40733 | 0,708197 | 0,38223 | 0,688771 |
| *PELI1* | 0,10780 | 0,207371 | 0,48667 | 0,781468 | 1,48353 | 0,700961 | 1,36299 | 0,805587 |
| *PIN1* | 0,85554 | 0,838942 | 0,47763 | 0,372677 | 3,20629 | 0,134954 | 0,99689 | 0,997143 |
| *PPC* | 1,07909 | 0,905309 | 0,77399 | 0,521443 | 1,11042 | 0,888638 | 1,05356 | 0,924620 |
| *RELA* | 5,70738 | 0,291704 | 2,88398 | 0,590283 | 271,63528 | 0,056380 | 0,58148 | 0,736601 |
| *RTC* | 1,11672 | 0,864970 | 0,84147 | 0,655309 | 1,15896 | 0,849994 | 1,07630 | 0,897030 |
| *SPP1* | 0,93299 | 0,925800 | 2,94467 | 0,336476 | 2,41597 | 0,045673 | 2,49118 | 0,353323 |
| *STAT1* | 1,89872 | 0,056354 | 1,87421 | 0,049002 | 0,74116 | 0,382293 | 0,80834 | 0,305230 |
| *SUGT1* | 0,52730 | 0,114557 | 0,96256 | 0,977345 | 1,67625 | 0,004887 | 3,57379 | 0,161835 |
| *TBK1* | 0,47532 | 0,280872 | 0,59588 | 0,698358 | 2,73510 | 0,233715 | 1,10190 | 0,911131 |
| *TICAM2* | 3,67750 | 0,560403 | 7,87471 | 0,374110 | 2,14658 | 0,403252 | 0,34889 | 0,616111 |
| *TKFC* | 3,05160 | 0,102124 | 1,29075 | 0,698058 | 6,76363 | 0,010283 | 0,71281 | 0,526859 |
| *TLR2* | 0,90383 | 0,674729 | 0,72908 | 0,093723 | 1,11603 | 0,620462 | 1,28406 | 0,333201 |
| *TLR3* | 0,77626 | 0,411215 | 0,79978 | 0,698959 | 1,55416 | 0,483509 | 1,13698 | 0,848369 |
| *TLR7* | 0,83403 | 0,799885 | 0,38576 | 0,162258 | 0,92751 | 0,909577 | 0,55185 | 0,544170 |
| *TLR8* | 1,54506 | 0,678740 | 1,76283 | 0,611745 | 1,06251 | 0,917151 | 0,56177 | 0,276220 |
| *TNF* | 3,89170 | 0,248507 | 3,33983 | 0,370752 | 6,89600 | 0,008439 | 0,81344 | 0,457066 |
| *TRAF3* | 0,95318 | 0,955945 | 1,16570 | 0,851420 | 0,73123 | 0,692609 | 0,48100 | 0,132187 |
| *TRAF6* | 0,85074 | 0,775876 | 0,84072 | 0,794055 | 8,65706 | 0,188120 | 4,90233 | 0,318750 |

Table S3. Fold regulation of genes, compared to untreated control (moMΦ), transformed in z-score values.

| | 3 h | | | | 24 h | | | |
|---|---|---|---|---|---|---|---|---|
| | IL-1β | | IL-1Ra | | IL-1β | | IL-1Ra | |
| Symbol | Fold-change | z-score | Fold-change | z-score | Fold-change | z-score | Fold-change | z-score |
| *ATG5* | 2,66425 | -0,03594 | 6,28601 | 3,67259 | 2,07783 | -0,40215 | 1,08257 | -0,24962 |
| *AZI2* | 25,79751 | 5,37561 | 3,25112 | 1,31644 | 54,15071 | 4,74555 | 1,05999 | -0,25447 |
| *BCL2* | 0,36931 | -0,57280 | 0,88415 | -0,52117 | 0,46327 | -0,56176 | 0,52608 | -0,36923 |
| *CASP10* | 2,85071 | 0,00768 | 2,12384 | 0,44127 | 0,78982 | -0,52948 | 1,91459 | -0,07079 |
| *CASP8* | 0,88113 | -0,45307 | 1,33829 | -0,16860 | 3,01371 | -0,30963 | 2,60666 | 0,07796 |
| *CCL4* | 4,24853 | 0,33467 | 1,77237 | 0,16841 | 1,7678 | -0,43280 | 0,58954 | -0,35559 |
| *CCL5* | 1,03599 | -0,41684 | 0,54203 | -0,78678 | 4,06613 | -0,20559 | 0,36462 | -0,40393 |
| *CD80* | 2,92086 | 0,02409 | 4,24835 | 2,09065 | 7,4465 | 0,12857 | 0,78946 | -0,31262 |
| *CD86* | 0,80181 | -0,47162 | 1,51912 | -0,02821 | 5,62252 | -0,05174 | 0,56141 | -0,36164 |
| *CHUK* | 1,85049 | -0,22631 | 0,90597 | -0,50423 | 2,97595 | -0,31337 | 0,74005 | -0,32324 |
| *CSF2* | 2,06284 | -0,17663 | 1,45111 | -0,08101 | 0,08477 | -0,59917 | 0,65358 | -0,34182 |
| *CTSB* | 1,97079 | -0,19816 | 1,07871 | -0,37012 | 4,36312 | -0,17624 | 1,21284 | -0,22162 |
| *CTSS* | 0,83732 | -0,46332 | 0,86486 | -0,53615 | 1,1331 | -0,49554 | 0,73832 | -0,32361 |
| *CTSV* | 0,7743 | -0,47806 | 0,55426 | -0,77728 | 3,76203 | -0,23566 | 4,53112 | 0,49159 |
| *CXCL10* | 1,26077 | -0,36426 | 0,5368 | -0,79084 | 3,65954 | -0,24579 | 3,05794 | 0,17496 |
| *CXCL11* | 2,73794 | -0,01870 | 1,26819 | -0,22302 | 9,79526 | 0,36076 | 0,97295 | -0,27318 |
| *CXCL8* | 0,92906 | -0,44185 | 0,85481 | -0,54395 | 10,28474 | 0,40915 | 5,10916 | 0,61583 |
| *CXCL9* | 3,79723 | 0,22909 | 1,3563 | -0,15461 | 13,75459 | 0,75216 | 0,94752 | -0,27865 |

| | | | | | | | | |
|---|---|---|---|---|---|---|---|---|
| *CXCR4* | 1,78516 | -0,24159 | 1,16878 | -0,30020 | 1,49298 | -0,45997 | 3,6954 | 0,31197 |
| *CYLD* | 2,11674 | -0,16402 | 1,15556 | -0,31046 | 4,35331 | -0,17721 | 1,13623 | -0,23809 |
| *DDX58* | 2,01609 | -0,18757 | 1,8967 | 0,26493 | 0,57155 | -0,55105 | 0,54389 | -0,36540 |
| *DHX58* | 6,24774 | 0,80234 | 0,23146 | -1,02789 | 4,84698 | -0,12840 | 0,61335 | -0,35047 |
| *FOS* | 2,14052 | -0,15846 | 0,64446 | -0,70725 | 30,85537 | 2,44267 | 2,65361 | 0,08805 |
| *HSP90AA1* | 1,4477 | -0,32053 | 1,89985 | 0,26738 | 4,74614 | -0,13837 | 1,11918 | -0,24175 |
| *IFIH1* | 0,96145 | -0,43428 | 0,79067 | -0,59374 | 2,86522 | -0,32431 | 1,83213 | -0,08851 |
| *IFNAR1* | 2,26313 | -0,12978 | 0,59521 | -0,74549 | 1,76402 | -0,43317 | 0,69001 | -0,33399 |
| *IFNB1* | 2,21324 | -0,14145 | 1,18812 | -0,28518 | 6,54445 | 0,03940 | 0,90485 | -0,28782 |
| *IKBKB* | 4,35135 | 0,35872 | 2,35916 | 0,62396 | 1,56585 | -0,45276 | 0,37106 | -0,40255 |
| *IL12A* | 2,05592 | -0,17825 | 4,62026 | 2,37938 | 0,34642 | -0,57331 | 4,62177 | 0,51108 |
| *IL15* | 1,38149 | -0,33602 | 1,25688 | -0,23180 | 2,22505 | -0,38760 | 1,77018 | -0,10183 |
| *IL18* | 7,22478 | 1,03090 | 1,17515 | -0,29525 | 13,50582 | 0,72757 | 0,07923 | -0,46527 |
| *IL1B* | 1,13827 | -0,39291 | 0,05001 | -1,16876 | 4,1859 | -0,19375 | 27,93615 | 5,52215 |
| *IL23A* | 1,99437 | -0,19265 | 1,09294 | -0,35907 | 4,02562 | -0,20960 | 0,85567 | -0,29839 |
| *IRAK1* | 0,72067 | -0,49060 | 2,30029 | 0,57826 | 1,66795 | -0,44267 | 1,00419 | -0,26647 |
| *IRF3* | 0,77699 | -0,47743 | 1,17904 | -0,29223 | 0,33157 | -0,57478 | 1,25275 | -0,21304 |
| *IRF5* | 1,6697 | -0,25354 | 2,13007 | 0,34812 | 0,53913 | -0,27605 | 0,51547 | -0,50397 |
| *JUN* | 99,29421 | 5,55112 | 3,24637 | 1,10024 | 518,66291 | 5,19189 | 0,3361 | -0,62887 |
| *LOC10052* | 0,65147 | -0,31408 | 0,21571 | -0,94171 | 0,62636 | -0,27523 | 0,7598 | -0,33384 |
| *LOC10073* | 4,5191 | -0,08412 | 2,72109 | 0,74633 | 3,08704 | -0,25208 | 0,80036 | -0,30560 |
| *MAP2K1* | 4,22272 | -0,10174 | 1,08685 | -0,35477 | 15,73131 | -0,13311 | 0,75763 | -0,33535 |
| *MAP3K1* | 14,06161 | 0,48327 | 2,54124 | 0,62515 | 31,24289 | 0,01285 | 0,37173 | -0,60406 |
| *MAP3K7* | 2,47859 | -0,20544 | 3,94567 | 1,57141 | 1,2265 | -0,26959 | 0,48803 | -0,52308 |
| *MAPK1* | 0,21842 | -0,33983 | 0,21475 | -0,94236 | 2,98824 | -0,25301 | 1,09834 | -0,09811 |
| *MAPK14* | 1,24482 | -0,27880 | 1,47258 | -0,09487 | 25,94504 | -0,03700 | 1,93201 | 0,48239 |
| *MAPK3* | 2,57939 | -0,19945 | 1,91943 | 0,20620 | 3,24462 | -0,25060 | 1,12142 | -0,08204 |
| *MAVS* | 0,33224 | -0,33306 | 0,3067 | -0,88040 | 8,17961 | -0,20416 | 7,51675 | 4,37113 |
| *MEFV* | 15,34682 | 0,55969 | 1,58134 | -0,02160 | 24,45742 | -0,05100 | 0,75424 | -0,33771 |
| *MX1* | 0,26934 | -0,33680 | 0,52183 | -0,73546 | 2,90595 | -0,25378 | 0,15015 | -0,75835 |
| *MYD88* | 2,54806 | -0,20131 | 0,98295 | -0,42477 | 12,03454 | -0,16789 | 0,96152 | -0,19338 |
| *NLRP3* | 15,226 | 0,55250 | 0,76487 | -0,57170 | 1,19739 | -0,26986 | 0,41662 | -0,57280 |
| *NOD2* | 13,31886 | 0,43911 | 2,16421 | 0,37112 | 14,22544 | -0,14727 | 1,17139 | -0,04724 |
| *OAS2* | 0,51731 | -0,32206 | 0,62286 | -0,66739 | 0,40733 | -0,27729 | 0,38223 | -0,59675 |
| *PELI1* | 0,1078 | -0,34641 | 0,48667 | -0,75915 | 1,48353 | -0,26717 | 1,36299 | 0,08617 |
| *PIN1* | 0,85554 | -0,30195 | 0,47763 | -0,76524 | 3,20629 | -0,25096 | 0,99689 | -0,16875 |
| *PPC* | 1,07909 | -0,28866 | 0,77399 | -0,56556 | 1,11042 | -0,27068 | 1,05356 | -0,12929 |
| *RELA* | 5,70738 | -0,01346 | 2,88398 | 0,85608 | 271,63528 | 2,27472 | 0,58148 | -0,45801 |
| *RTC* | 1,11672 | -0,28642 | 0,84147 | -0,52009 | 1,15896 | -0,27022 | 1,0763 | -0,11346 |
| *SPP1* | 0,93299 | -0,29734 | 2,94467 | 0,89697 | 2,41597 | -0,25839 | 2,49118 | 0,87175 |
| *STAT1* | 1,89872 | -0,23992 | 1,87421 | 0,17573 | 0,74116 | -0,27415 | 0,80834 | -0,30004 |
| *SUGT1* | 0,5273 | -0,32147 | 0,96256 | -0,43851 | 1,67625 | -0,26535 | 3,57379 | 1,62559 |
| *TBK1* | 0,47532 | -0,32456 | 0,59588 | -0,68556 | 2,7351 | -0,25539 | 1,1019 | -0,09563 |
| *TICAM2* | 3,6775 | -0,13416 | 7,87471 | 4,21866 | 2,14658 | -0,26093 | 0,34889 | -0,61996 |
| *TKFC* | 3,0516 | -0,17137 | 1,29075 | -0,21739 | 6,76363 | -0,21749 | 0,71281 | -0,36656 |
| *TLR2* | 0,90383 | -0,29908 | 0,72908 | -0,59582 | 1,11603 | -0,27063 | 1,28406 | 0,03121 |
| *TLR3* | 0,77626 | -0,30666 | 0,79978 | -0,54818 | 1,55416 | -0,26650 | 1,13698 | -0,07120 |
| *TLR7* | 0,83403 | -0,30323 | 0,38576 | -0,82714 | 0,92751 | -0,27240 | 0,55185 | -0,47864 |
| *TLR8* | 1,54506 | -0,26095 | 1,76283 | 0,10069 | 1,06251 | -0,27113 | 0,56177 | -0,47173 |
| *TNF* | 3,8917 | -0,12142 | 3,33983 | 1,16321 | 6,896 | -0,21624 | 0,81344 | -0,29649 |
| *TRAF3* | 0,95318 | -0,29614 | 1,1657 | -0,30164 | 0,73123 | -0,27425 | 0,481 | -0,52797 |
| *TRAF6* | 0,85074 | -0,30223 | 0,84072 | -0,52060 | 8,65706 | -0,19967 | 4,90233 | 2,55067 |

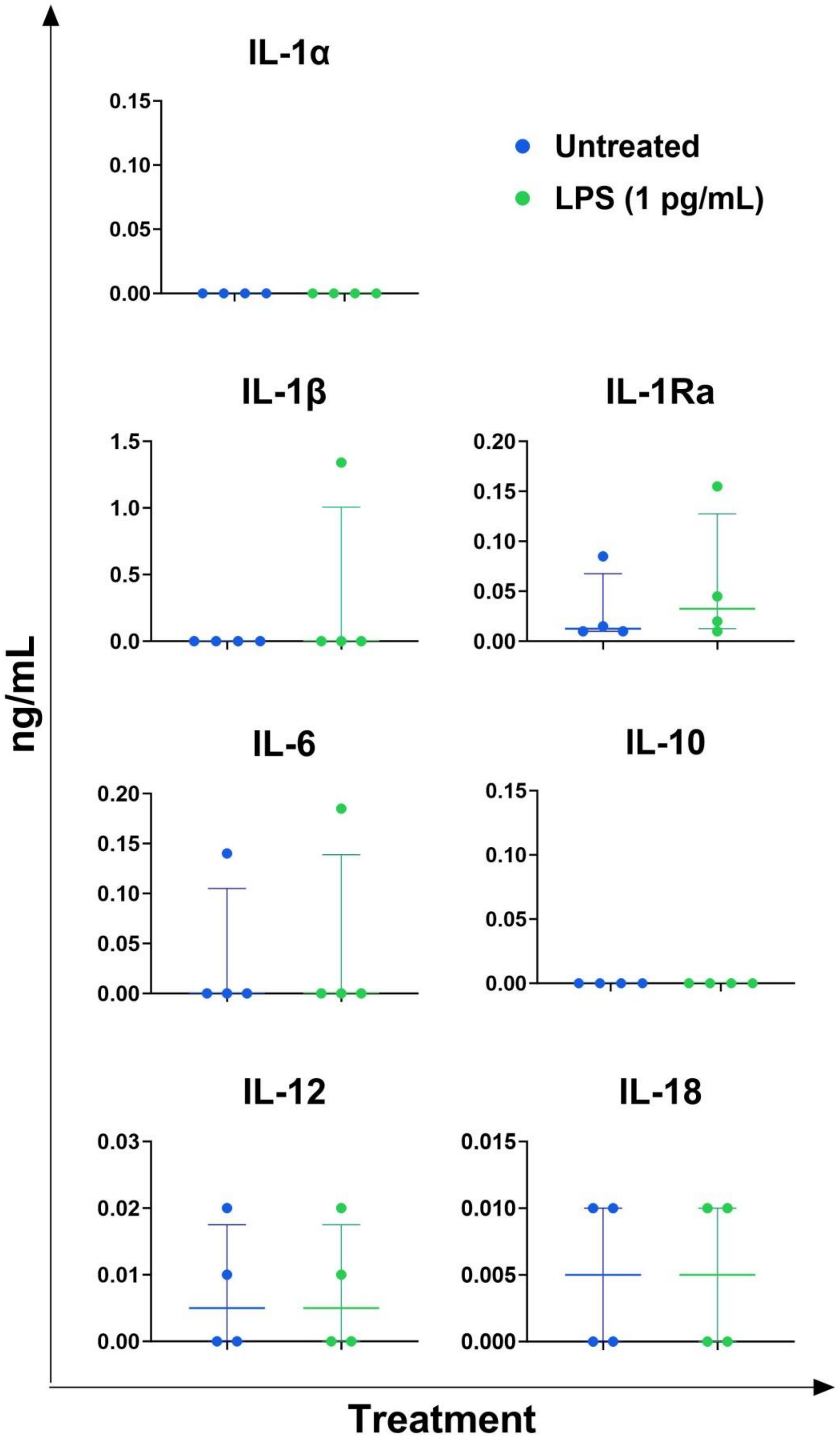


**Figure S1. Cytokine release by porcine moMΦ in response to stimulation with 1 pg/mL of LPS.** Porcine moMΦ were left untreated or stimulated with 1 pg/mL of LPS. After 24h, levels of IL-1α, IL-1β, IL1Ra, IL-6, IL-10, IL-12, and IL-18 was quantified with multiplex ELISA. Data from diverse animals are presented. Differences between treated and untreated cells were evaluated with the Wilcoxon test.

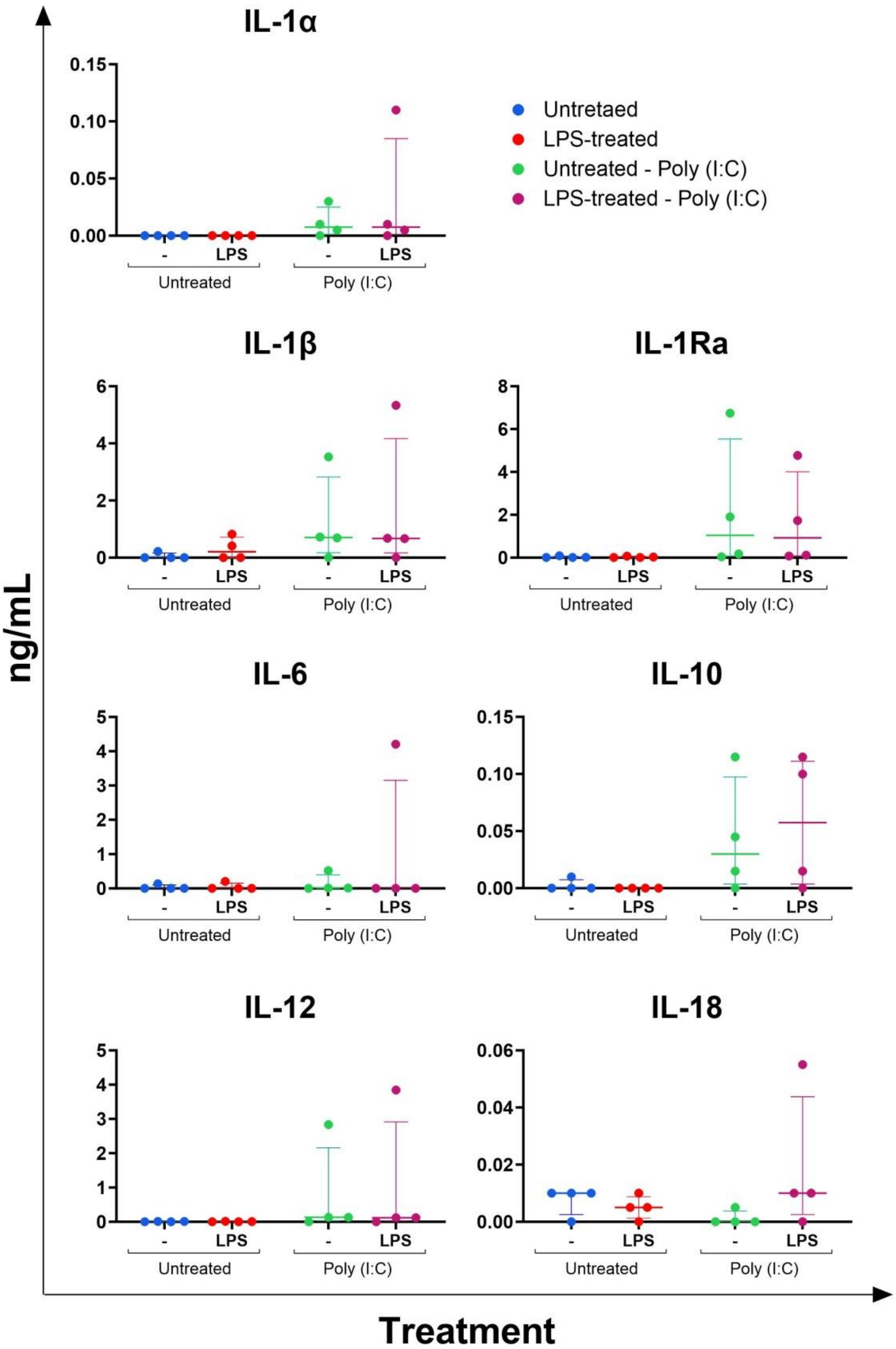


**Figure S2. Impact of pre-treatement with LPS (0.5 pg/ml) on porcine moMΦ response to Poly (I:C) stimulation.** Porcine moMΦ were left untreated or stimulated with 0.5 pg/mL of LPS. After 24h, supernantants was removed and replaced with fresh cRMPI ('untreated') or cRMPI supplemented with Poly (I:C). 24 h later, levels of IL-1α, IL-1β, IL1Ra, IL-6, IL-10, IL-12, and IL-18 was quantified with multiplex ELISA. Data from diverse animals are presented. For each condition (untreated and Poly (I:C), differences between LPS-treated and untreated cells ('-') were evaluated with the Wilcoxon test.

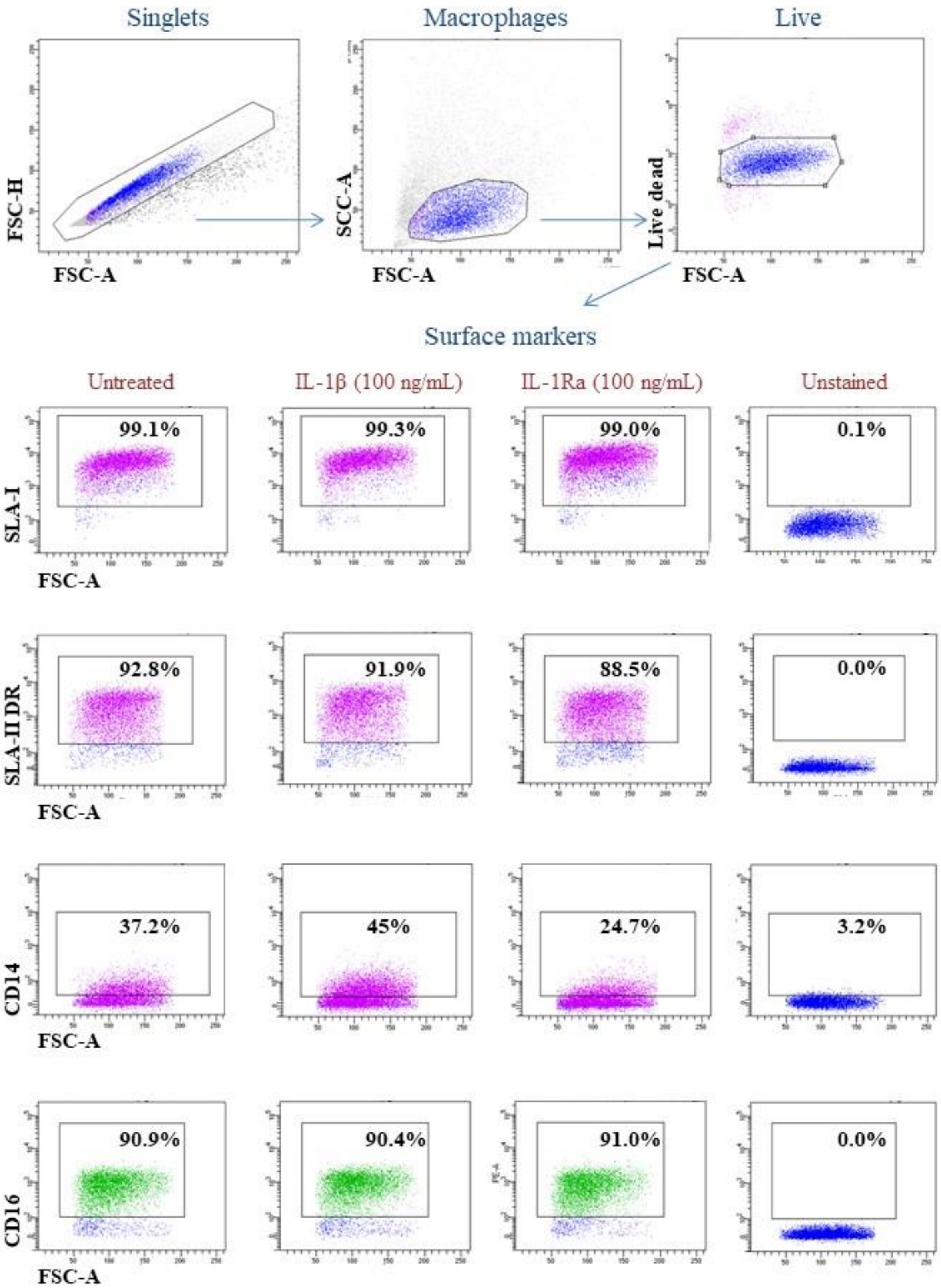


**Figure S3. Gating strategy used to evaluate IL-1β and IL-1Ra modulation on surface markers.** Porcine macrophages were left untreated of stimulated with recombinant IL-1β or IL-1Ra. 24 h post-stimulation, dimension (FSC-A), granularity (SCC-A) and surface expression of SLA class I, SLA class II DR, CD14, CD16 were investigated using flow cytometry. On the top, gating strategy used to investigate surface marker expression is presented: exclusion of doublets, gating on viable moMΦ, then assessing the staining for surface markers. Below, for each markes, representative dot plots of untreated (moMΦ), IL-1β-treated moMΦ, and IL-1Ra-treated moMΦ are presented, alongside unstained controls.

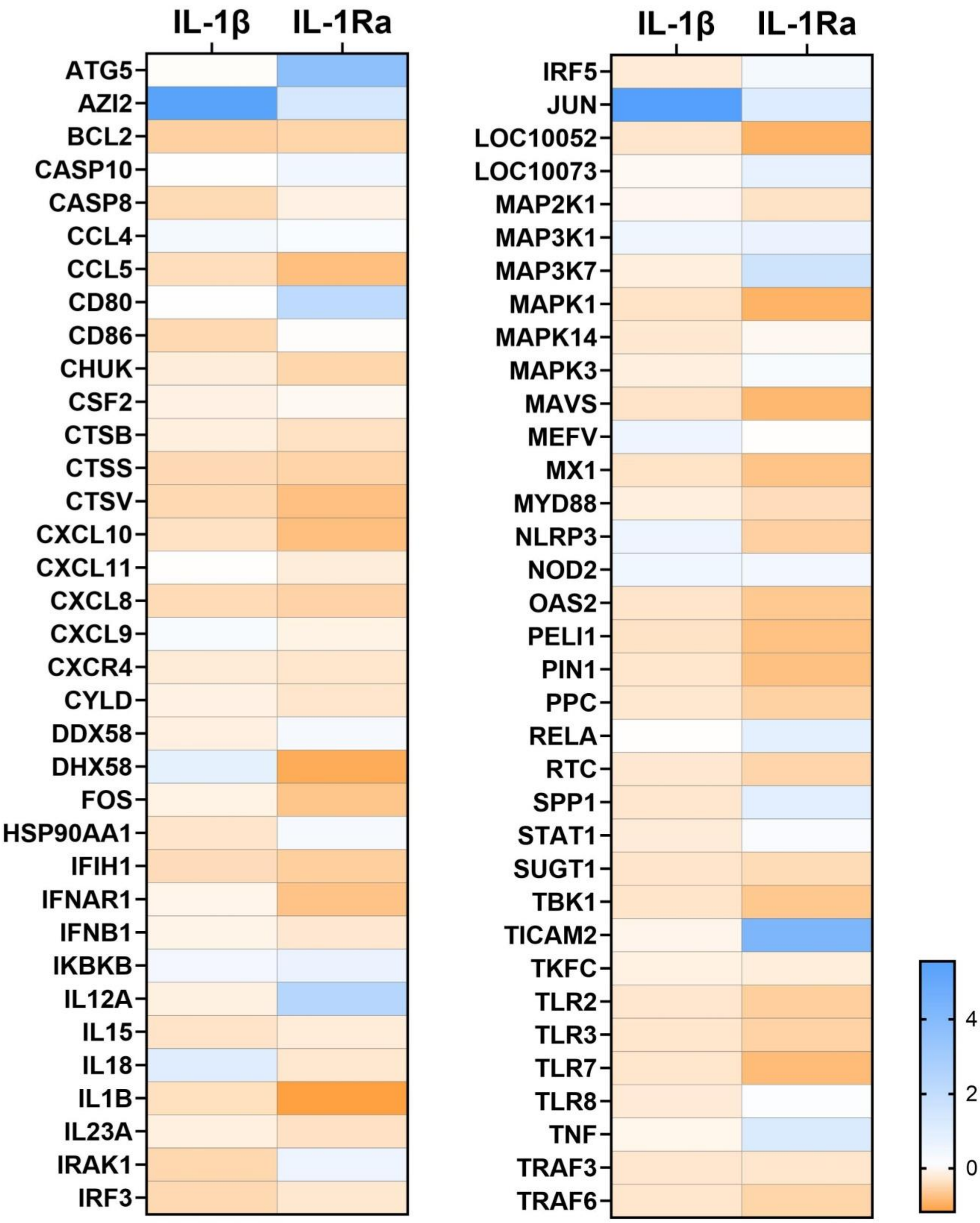


**Figure S4. Z-score values of 70 antiviral genes in porcine moMΦ 3 h post-stimulation with IL-1β and IL-1Ra.** Porcine moMΦ were left untreated, or they were stimulated with IL-1β (100 ng/mL), or IL-1Ra (100 ng/mL). At 3h post-stimulation, macrophages were analyzed using the RT2 Profiler PCR Array for 70 antiviral genes. The heatmap illustrates z-score values of fold change expression of immune-related genes. Data of four independent experiments utilizing different blood donors are presented. Colours represent the intensity of z-score values: blue represents the highest value, white the baseline value (0), and orange the smallest value.

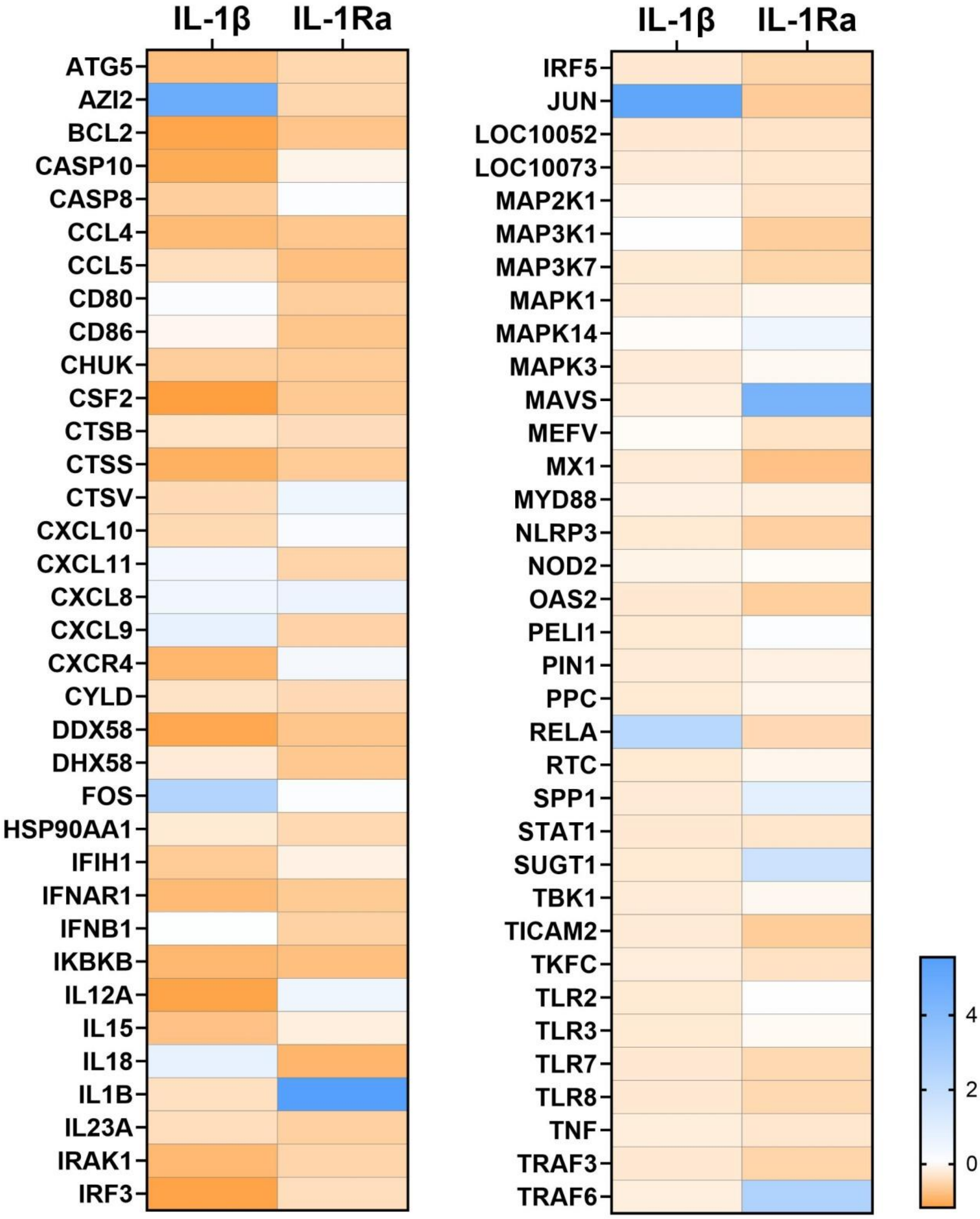


**Figure S5. Z-score values of 70 antiviral genes in porcine moMΦ 24 h post-stimulation with IL-1β and IL-1Ra.** Porcine moMΦ were left untreated, or they were stimulated with IL-1β (100 ng/mL), or IL-1Ra (100 ng/mL). At 24h post-stimulation, macrophages were analyzed using the RT2 Profiler PCR Array for 70 antiviral genes. The heatmap illustrates z-score values of fold change expression of immune-related genes. Data of four independent experiments utilizing different blood donors are presented. Colours represent the intensity of z-score values: blue represents the highest value, white the baseline value (0), and orange the smallest value.
